# *Helicobacter pylori* Outer Membrane Vesicles Engage TLR2 Through NapA to Promote Epithelial Association and Inflammatory Signalling

**DOI:** 10.1002/jev2.70376

**Published:** 2026-09-28

**Authors:** Mariaignacia Rubilar, Rosy Brito, Annegrett Palavecino, Joan Villena, Marco Mellado, María I. Silva, Manuel Valenzuela‐Valderrama

**Affiliations:** ^1^ Laboratorio de Carcinogénesis Molecular, Instituto de Investigación y Doctorados Universidad Central de Chile Santiago Chile; ^2^ Centro Interdisciplinario de Investigación Biomédica e Ingeniería para la Salud (MEDING), Facultad de Medicina, Campus de la Salud Universidad de Valparaíso Viña del Mar Chile; ^3^ Dirección de Investigación Universidad Bernardo O'Higgins Santiago Chile; ^4^ Laboratorio de Espectroscopía y Química Aplicada Instituto de Investigación y Doctorados Universidad Central de Chile Santiago Chile

**Keywords:** *Helicobacter pylori*, inflammation, NapA, NF‐κB, OMV, PAMPs, sulforaphane, Toll‐like receptor 2, urease

## Abstract

*Helicobacter pylori* infection is characterized by chronic gastric inflammation driven by persistent activation of innate immune signalling pathways. Outer membrane vesicles (OMVs) released by *H. pylori* are important mediators of host–pathogen interactions. Although several mechanisms of OMV internalization have been described, how specific OMV‐associated bacterial factors engage host innate immune receptors and couple vesicle association to inflammatory signalling remains poorly understood. Here, we investigated the contribution of OMV‐associated virulence factors and Toll‐like receptor 2 (TLR2) heterodimers to OMV–host cell interactions in gastric epithelial cells. Using engineered AGS and GES‐1 reporter cells expressing TLR2/1 or TLR2/6, together with genetically defined OMVs, we found that *H. pylori* OMVs preferentially associated with TLR2‐expressing cells and induced robust NF‐κB activation. Comparative analysis of OMVs from wild‐type and isogenic mutant strains identified Neutrophil‐Activating Protein A (NapA) as a major determinant of TLR2‐dependent OMV association and inflammatory signalling, whereas urease had a more limited contribution. Disruption of NapA or neutralization of TLR2 markedly reduced OMV association with gastric epithelial cells. Purified *H. pylori* lipopolysaccharide elicited a comparatively weak and TLR2/6‐biased response, indicating that it does not account for the robust TLR2‐dependent signalling induced by intact OMVs. Furthermore, OMV‐associated NapA exhibited greater pro‐inflammatory activity than soluble recombinant NapA, supporting a role for the vesicular context in enhancing TLR2‐dependent signalling. Pharmacological inhibition with sulforaphane significantly attenuated OMV‐induced TLR2/NF‐κB activation. Collectively, these findings identify a previously unrecognized role for OMV‐associated NapA in linking TLR2‐dependent vesicle association with gastric epithelial cells to inflammatory signalling and support a model in which vesicular presentation

## 1 Introduction

*Helicobacter pylori* (*H. pylori*) colonizes the gastric epithelium in nearly 50% of the global population, with higher prevalence in developing regions. The bacterium is typically acquired in childhood and persists lifelong without eradication therapy (Martin‐Nunez et al. 2019). Although most infections remain asymptomatic due to long‐term host–microbe co‐evolution (Atherton and Blaser 2009), host genetic susceptibility and bacterial virulence determinants can disrupt this equilibrium, predisposing some individuals to severe gastric and duodenal diseases (Bravo et al. 2018). Beyond the stomach, accumulating evidence links *H. pylori* infection to several extra‐gastric conditions, including certain neurodegenerative disorders (Bravo et al. 2018; Doulberis et al. 2018; Contaldi et al. 2017; Palacios et al. 2023).

*H. pylori* is classified as a Group I carcinogen (IARC 1994) because chronic infection markedly increases the risk of gastric cancer (GC) (Polk and Peek 2010; West et al. 2017; Sukri et al. 2020). The intestinal type accounts for the majority of cases (West et al. 2017; Zou et al. 2017) and typically progresses through the Correa cascade, from chronic gastritis to atrophy, intestinal metaplasia, dysplasia and ultimately adenocarcinoma (Shimizu et al. 2014; Correa and Piazuelo 2012). Persistent inflammation is a key driver of this process (Valenzuela et al. 2015; Echizen et al. 2016). During *H. pylori*–associated carcinogenesis, a predominant Th1 response leads to sustained production of proinflammatory cytokines, including IL‐8, IL‐6, IL‐1β and TNF (Correa and Piazuelo 2012; Valenzuela et al. 2015). Host cytokine gene polymorphisms, together with infection by virulent strains, strongly influence disease outcome (Valenzuela et al. 2015). These interactions have been recapitulated in murine models that reproduce the human cascade (Asano et al. 2016; Huang et al. 2013; Thalmaier et al. 2002; Li et al. 2006; Ogura et al. 2000). This inflammatory environment promotes the loss of specialized gastric cells and the emergence of progenitor‐like or metaplastic cell types (Valenzuela et al. 2015; Houghton et al. 2004).

Toll‐like receptor 2 (TLR2) is a pattern recognition receptor that detects diverse microbial ligands—including lipopeptides, peptidoglycan and lipoteichoic acid, as well as synthetic agonists such as Pam2CSK4, Pam3CSK4, CU‐T12‐9 and FSL‐1—and also host‐derived DAMPs (West et al. 2017; Kawai and Akira 2010). TLR2 has been implicated in pro‐tumorigenic signalling in human GC (West et al. 2017), and transgenic mouse models, such as gp130/STAT3 mice, have demonstrated a functional role for the TLR2/MyD88 axis in gastric tumorigenesis (Kennedy et al. 2014). TLR2 functions in combination with TLR1, TLR6 or TLR10 (Kawai and Akira 2010). Notably, TLR10 can act as an inhibitory modulator of TLR2‐mediated inflammation and is predominantly expressed in monocytes (Fore et al. 2020; Oosting et al. 2014). Regardless of whether inputs are activating or inhibitory, TLR2 signalling converges on MyD88 to activate NF‐κB, a transcription factor essential for gastric inflammation and cancer development (Sokolova and Naumann 2017). Although NF‐κB is crucial for immune homeostasis (Sue et al. 2015; Pahl 1999), it becomes constitutively active in many cancers and chronic inflammatory disorders (Xiao 2004).

The canonical NF‐κB pathway is initiated by cytokines, PAMPs and DAMPs through TNF receptors and TLRs (Taniguchi and Karin 2018). Signal transduction via TRAF proteins, TRIF and RIP kinases activates the IKK complex, composed of IKKα, IKKβ and the regulatory subunit NEMO (Taniguchi and Karin 2018). IKK‐mediated phosphorylation and degradation of IκBα enable NF‐κB dimers, such as the canonical p50/p65, to translocate to the nucleus and drive transcription of genes involved in cytokine production, cell adhesion, apoptosis regulation and broader inflammatory responses (Xiao 2004; Taniguchi and Karin 2018; Gilmore 2006; Viatour et al. 2005).

*H. pylori* is considered poorly invasive, residing mainly within the superficial gastric mucosa. However, individual bacteria can occasionally be detected inside epithelial cells, in intercellular spaces, or within the lamina propria, in proximity to immune‐inflammatory cells (Fiocca et al. 1999; Necchi et al. 2007). Increasing evidence highlights the importance of outer membrane vesicles (OMVs) in bacterial pathogenesis (Chen et al. 2026). Similar to other Gram‐negative species, *H. pylori* continuously releases OMVs during growth in vitro and in vivo (Turner et al. 2018). Early studies proposed that OMVs contribute to the progression of *H. pylori*–associated gastric diseases, including GC (Fiocca et al. 1999). OMVs have therefore been suggested to act as virulence‐enhancing elements that extend the pathogenic capacity of this otherwise poorly invasive bacterium (Fiocca et al. 1999; Chmiela et al. 2018). Consistent with this, OMVs have been identified in the stomachs of infected patients (West et al. 2017) and exhibit morphologies and compositions similar to those of OMVs isolated from cultured *H. pylori* (Fiocca et al. 1999).

The interaction of *H. pylori* OMVs with gastric epithelial cells involves an initial association with the cell surface followed by vesicle internalization. Previous studies have shown that *H. pylori* OMVs can enter epithelial cells through multiple endocytic routes, including clathrin‐dependent endocytosis, caveolin‐mediated uptake and macropinocytosis, with the relative contribution of these pathways influenced by vesicle size and composition (Turner et al. 2018). Vesicle‐associated factors such as VacA have also been reported to influence OMV association with gastric epithelial cells (Parker et al. 2010). Thus, while the cellular mechanisms involved in OMV uptake have been increasingly characterized, the bacterial determinants that mediate the initial interaction of OMVs with host innate immune receptors and connect vesicle association to inflammatory signalling remain poorly defined.

*H. pylori* OMVs carry multiple virulence factors, including abundant surface‐associated urease (Palacios et al. 2023), and exhibit diverse biological activities. However, studies evaluating their ability to activate NF‐κB remain limited. Choi et al. reported NF‐κB activation and IL‐8 secretion in epithelial cells exposed to OMVs from clinical isolates (Choi et al. 2021). Kaparakis et al. showed that OMV‐associated peptidoglycan from several Gram‐negative bacteria, including *H. pylori*, activates NF‐κB via NOD1 (Kaparakis et al. 2010). Our group has recently demonstrated that OMVs from *H. pylori* activate NF‐κB in astrocytes, contributing to neuronal damage in vitro and in vivo (Palacios et al. 2023). Among OMV‐associated proteins, neutrophil‐activating protein A (NapA) has emerged as an important immunomodulatory factor of *H. pylori* (Fu 2014, Wang et al. 2006) and a recurrent component of *H. pylori* OMV cargo (Turner et al. 2018; Mullaney et al. 2009; Palacios et al. 2023). Beyond its established role as a ferritin‐like protein involved in oxidative stress resistance and bacterial colonization (Satin et al. 2000; Fu and Lai 2022), NapA has been reported to promote inflammatory responses and activate innate immune signalling pathways, including TLR2‐dependent signalling (Fu 2014; Satin et al. 2000; Wen et al. 2021; Codolo et al. 2022). However, whether OMV‐associated NapA contributes to TLR2‐dependent NF‐κB activation in gastric epithelial cells remains unknown.

Despite increasing recognition of *H. pylori* OMVs as mediators of host–pathogen communication, the mechanisms by which specific OMV‐associated components promote TLR2‐dependent signalling in gastric epithelial cells remain incompletely understood. In particular, it is unclear whether OMV‐induced inflammatory responses reflect passive delivery of bacterial components or instead rely on defined OMV‐associated determinants that promote receptor engagement and signalling specificity. In this study, we investigated the capacity of *H. pylori* OMVs to activate NF‐κB in gastric epithelial cells and dissected the contribution of TLR2 heterodimers and individual OMV‐associated virulence factors, including NapA, urease, VacA, CagA and lipopolysaccharide. By combining genetic, biochemical and functional approaches, we aimed to define the molecular basis of OMV–host cell interactions and identify major OMV‐associated determinants that contribute to TLR2‐dependent inflammatory signalling.

## 2 Materials and Methods

### 2.1 Cell Lines and Culture Conditions

The human gastric cancer‐derived cell lines AGS (ATCC CRL‐1739), MKN45 (JCRB0254) and MKN74 (JCRB0255), as well as the immortalized human gastric epithelial cell line GES‐1 (kindly provided by Dr. Dawit Kidane, The University of Texas at Austin, USA), were cultured in RPMI 1640 medium (Thermo Fisher Scientific) supplemented with 2 mM glutamine, 10% fetal bovine serum (Cytiva) and antibiotics (100 U/mL penicillin and 100 µg/mL streptomycin), and maintained at 37°C in a humidified incubator with 5% CO_2_.

### 2.2 *H. pylori* Strains and Culture Conditions

*H. pylori* strains 26695 (ATCC 700392) and 60190 (ATCC 49503), as well as the isogenic mutants 26695 Δ*cagA* and 26695 Δ*vacA*, were kindly provided by Dr. Guillermo Pérez‐Pérez (Adjunct Professor, New York University). Bacterial strains were routinely cultured on trypticase soy agar (TSA) plates supplemented with 5% horse donor serum (PAN Biotech), Vitox supplement and Dent selective supplement (Oxoid Limited, Basingstoke, UK). Cultures were incubated at 37°C for 24 h under microaerophilic conditions (5% CO_2_ and high humidity). Isogenic *H. pylori* 26695 Δ*napA*, Δ*waaL* (HP1039) and Δ*lex2B* (HP0826) (Teng et al. 2022), as well as *H. pylori* 60190 Δ*ure* mutants, were generated by allelic exchange using interrupted alleles containing a chloramphenicol acetyltransferase (CAT) resistance cassette, as previously described (Valenzuela‐Valderrama et al. 2019). The *H. pylori* 26695 Δ*ure* mutant was constructed in our previous study (Valenzuela‐Valderrama et al. 2019). All mutant strains were confirmed by PCR and further characterized by Western blotting and SDS‐PAGE, followed by Coomassie blue or silver nitrate staining, as appropriate.

### 2.3 Obtention of TLR2/NF‐κB Reporter Cells

Gastric epithelial cells were transfected with the NanoLuc reporter vector containing a canonical NF‐κB response element (pNL3.2.NF‐κB‐RE[NlucP/NF‐κB‐RE/Hygro], Promega) using ViaFect (Promega). The following day, cells were selected with hygromycin (300 µg/mL) until resistant colonies were established. NF‐κB reporter activity was verified by stimulating cells with TNF (20 ng/mL, R&D Systems) for 5 h, followed by cell lysis and measurement of NanoLuc luciferase activity using the Nano‐Glo Luciferase Assay System (Promega) on a TECAN Infinite M200 PRO plate reader according to the manufacturer's instructions. A pool of NF‐κB reporter cells (derived from three independent clones) was then transfected with either pDuo‐TLR2/1, pDuo‐TLR2/6 or the control pDuo‐MCS plasmid (InvivoGen) and selected with blasticidin (10 µg/mL) for 6 days. Individual clones were isolated, and TLR1, TLR2 and TLR6 expression was confirmed by Western blot. To validate the responsiveness of the reporter clones, TLR2/1–NF‐κB cells were stimulated with Pam3CSK4 (InvivoGen) and CU‐T12‐9 (Tocris), and TLR2/6–NF‐κB cells were stimulated with Pam2CSK4 and FSL‐1 (InvivoGen).

### 2.4 *H. pylori* OMVs Purification

OMVs from *H. pylori* strains 26695 and 60190 were purified as previously described by our group (Palacios et al. 2023). Briefly, bacteria were cultured in Brucella broth supplemented with 0.25% β‐cyclodextrin (Sigma‐Aldrich), Dent selective supplement and Isovitalex (Oxoid). Cultures were centrifuged at 4500 rpm for 20 min, and supernatants were filtered through 0.2 µm filters to remove bacterial cells. The filtrates were concentrated using Amicon centrifugal filters with a 100 kDa molecular weight cut‐off (MWCO) (Sigma‐Aldrich). OMVs were precipitated by overnight incubation with Exo‐spin precipitation buffer EXO 01 (Cell Guidance Systems) at 4°C, followed by centrifugation at 11,000 × *g* for 1 h at 4°C. Pellets were resuspended in 500 µL PBS and desalted using a Vivaspin 2.5 MWCO column (Cytiva). Protein concentration was determined using the Pierce BCA Protein Assay Kit (Thermo Fisher Scientific). OMV preparations were aliquoted at 1 mg/mL and stored at –80°C until use. OMV size distribution and particle concentration were assessed by nanoparticle tracking analysis (NTA) using a NanoSight NS300 (Malvern Panalytical) following the manufacturer's recommendations. OMV morphology was evaluated by transmission electron microscopy (TEM) using a Talos F200C transmission electron microscope (Thermo Fisher Scientific). All OMV preparations used in this study were isolated and normalized using the same standardized protocol and characterized by nanoparticle tracking analysis, which revealed comparable size distributions and particle concentrations across strains.

### 2.5 LPS Purification and Quantification

LPS from *H. pylori* strain 26695 was extracted from bacterial pellets obtained from exponential‐phase liquid cultures grown in Brucella broth supplemented with 0.25% β‐cyclodextrin, Dent selective supplement, and Isovitalex under microaerophilic conditions using the hot phenol–water method (Westphal and Jann 1965). The extracted LPS was sequentially treated with DNase I, RNase A and proteinase K, followed by a second hot phenol extraction. The preparation was subsequently desalted using Zeba Spin Desalting Columns (7K MWCO; Thermo Fisher Scientific), precipitated with ethanol, and resuspended in molecular‐grade water. LPS concentration was determined by quantification of 2‐keto‐3‐deoxyoctonate (Kdo) using the butanol extraction assay described by Lam et al. (2014). A standard curve was generated using commercially available 2‐keto‐3‐deoxyoctonate ammonium salt (Sigma‐Aldrich). For stimulation experiments comparing purified *H. pylori* 26695 and *Escherichia coli* O55:B5 LPS (Sigma‐Aldrich), preparations were normalized according to their Kdo content, and equivalent Kdo concentrations were used in all assays.

### 2.6 TLR2–NF‐κB Reporter Activity

AGS and GES‐1 NF‐κB reporter cells were seeded at 10,000 cells per well in 96‐well plates and cultured for 24 h under standard conditions. Cells were then stimulated for 5 h with: (i) TLR2/1 agonists (CU‐T12‐9 and Pam3CSK4) at 1 nM–10 µM (CU‐T12‐9) or 1 ng/mL–10 µg/mL (Pam3CSK4); (ii) TLR2/6 agonists (Pam2CSK4 and FSL‐1) at 1 ng/mL–10 µg/mL; and (iii) *H. pylori* OMVs at 5–100 µg/mL. TNF (20 ng/mL) and *E. coli* O55:B5 LPS (Sigma‐Aldrich) were used as positive controls for NF‐κB activation. After stimulation, cells were lysed, and NanoLuc luciferase activity was quantified using the Nano‐Glo Luciferase Assay System (Promega) according to the manufacturer's instructions. Given the short stimulation time (5 h), no loss of cell viability was observed.

### 2.7 Western Blot Analysis

Protein extracts were prepared in lysis buffer containing 20 mM HEPES (pH 7.4), 0.1% SDS, 0.05% NP‐40, and 1× complete protease inhibitor cocktail (Roche), as previously described (Palacios et al. 2023). Protein concentrations were determined using the Pierce BCA Protein Assay (Thermo Fisher Scientific) according to the manufacturer's instructions. Equal amounts of protein (60 µg per lane) were resolved by SDS–PAGE on 10% mini‐gels (Bio‐Rad) and transferred to nitrocellulose membranes. Membranes were blocked with 5% milk in PBS–0.1% Tween‐20 and incubated with the following primary antibodies: mouse monoclonal anti‐TLR1 (sc‐514399, 1:3,000), anti‐TLR2 (sc‐166900, 1:3,000), anti‐TLR4 (sc‐293072, 1:3,000) and anti‐*H. pylori* OMP (sc‐57779, 1:3,000) were from Santa Cruz Biotechnology; rabbit monoclonal anti‐TLR6 (#12717, 1:3,000), anti‐NF‐κB p50 (#3035, 1:3,000) and NF‐κB p65 (#8242, 1:3,000) were from Cell Signaling; rabbit polyclonal anti‐UreB (ab127916, 1:15,000) from Abcam, mouse monoclonal anti‐β‐actin (#A5316, 1:15,000) from Sigma‐Aldrich and rabbit monoclonal anti‐NapA (#32344, 1:2,000) from Cayman Chemical Company. Bound antibodies were detected using HRP‐conjugated donkey anti‐mouse (1:5,000, Invitrogen #SA1‐100) or goat anti‐rabbit (1:5,000, Thermo Fisher Scientific #31462) secondary antibodies. Signal development was performed using EZ‐ECL reagent (Biological Industries), and images were acquired with an ImageQuant LAS500 imaging system (GE Healthcare).

### 2.8 Neutralization Assays

TLR2–NF‐κB reporter cells (TLR2/1 and TLR2/6) were seeded at 10,000 cells per well in 96‐well plates and cultured for 24 h. The following day, the medium was removed, and 50 µL of neutralizing antibodies (2 µg/mL) against human TLR1 (mabg‐htlr1, InvivoGen), TLR2 (maba2‐htlr2, InvivoGen) or TLR6 (mabg‐htlr6, InvivoGen) were added to each well. Negative‐control isotype antibodies (IgA2 isotype, maba2‐ctrl or IgG1 isotype, mabg1‐ctlm; InvivoGen) were included in parallel. After 1 h of antibody incubation, 50 µL of *H. pylori* OMVs (20 or 40 µg/mL) were added to each well. Following a 5 h stimulation period, cells were lysed, and Nano‐Glo luminescence was quantified according to the manufacturer's instructions.

### 2.9 Reverse Transcription and Polymerase Chain Reaction

Quantitative RT‐qPCR analysis of proinflammatory cytokine mRNA levels was performed as previously described (Ren et al. 2017). Briefly, gastric cells were seeded at 8 × 10^5^ cells per well in 6 cm plates. After 24 h of culture, cells were treated with TNF (20 ng/mL) or *H. pylori* OMVs (10 µg/mL) for 5 h. Total RNA was extracted using TRIzol reagent (Invitrogen, USA), precipitated with 50% isopropanol, and washed with 75% ethanol. The RNA pellet was resuspended in nuclease‐free water to a concentration of 0.5 µg/µL. For cDNA synthesis, 1 µg of total RNA was used with the 5X All‐In‐One RT Mastermix (Applied Biological Materials, #G592). Quantitative PCR amplification was performed using Takyon Low ROX SYBR 2X MasterMix blue dTTP (Eurogentec) with specific primers for IL‐8, TNF‐α and GAPDH (Ren et al. 2017). qPCR was run on a QuantStudio 5 thermocycler (Thermo Fisher Scientific). Relative mRNA expression changes were calculated using the 2^−ΔΔCT^ method (Livak and Schmittgen 2001).

### 2.10 His‐tag Recombinant Proteins

For the obtention of recombinant UreA, UreB and NapA, chromosomal DNA from the *H. pylori* 26695 strain (ATCC 700392) was purified and amplified by PCR (Tm 58°C) using primer pairs Forward 5′‐ccgcatatgaaactcaccccaaaagag‐3′ and reverse 5′‐ccggtcgacctccttaattgtttttacatag‐3′ for UreA, Forward 5′‐ccgcatatgaaaaagattagcagaaaag‐3′ and Reverse 5′‐ccggtcgacgaaaatgctaaagagttgcgcc‐3′ for UreB, Forward 5′‐ccgcatatgatgaaaacatttgaaattct‐3′ and Reverse 5′‐ccggtcgacagccaaatgggcttgcagca‐3′ for NapA. Then, the fragments were digested with NdeI and SalI (Promega) and ligated into the pET21a+ plasmid (Sigma‐Aldrich). Recombinant plasmids were isolated from *E. coli* DH5α ampicillin‐resistant clones, and the identity of DNA inserts was verified by sequencing. Next, pet21a‐UreA, pet21a‐UreB and pet21a‐NapA plasmids were introduced into *E. coli* BL21 (DE3) strains, and expression was induced with IPTG 1 mM for 16 h. Bacterial pellets were resuspended in 10 mL of lysis/binding buffer (20 mM sodium phosphate, 300 mM NaCl, pH 7.4) and incubated in the presence of lysozyme (800 µg/mL) for 30 min. Bacteria were disrupted by sonication on ice, using six 10‐second bursts at high intensity with a 10‐second cooling period, for a total of 20 min. Lysates were cleared by centrifugation (12,000 × *g*, 15 min) and filtered (0.2 µm). The supernatants were added to HisPur Ni‐NTA Chromatography Cartridges (Thermo Fischer Scientific), washed three times with wash buffer (20 mM sodium phosphate, 300 mM NaCl, 5 mM Imidazole, pH 7.4), and His‐tag recombinant proteins were eluted with elution buffer (20 mM sodium phosphate, 300 mM NaCl, 500 mM Imidazole, pH 7.4). Samples were desalted using Vivaspin 500 centrifugal concentrators with a 3 kDa molecular weight cutoff (MWCO; Sigma‐Aldrich). The identity of recombinant proteins was examined by polyacrylamide gel electrophoresis, followed by Coomassie blue staining and immunoblotting with a mouse monoclonal anti‐His tag antibody (R&D Systems #MAB050).

### 2.11 Fluorescent Labelling and Analysis of OMV Association With Gastric Cells

OMVs derived from *H. pylori* wild‐type strain 26695 and its isogenic Δ*napA* mutant were isolated and fluorescently labelled with DiR (DiIC18(7); Invitrogen) as previously described (Palacios et al. 2023). AGS reporter cells were seeded onto glass coverslips and cultured for 24 h before stimulation. Cells were then incubated with DiR‐labelled OMVs (10 µg/mL) derived from either the WT or Δ*napA* strain for 5 h at 37°C. Following incubation, cells were extensively washed with PBS to remove non‐associated OMVs, fixed with 4% paraformaldehyde for 10 min at room temperature, and counterstained with DAPI. Coverslips were mounted in Fluoromount‐G (Invitrogen), and fluorescence images were acquired with an Axioscope 5 epifluorescence microscope (Zeiss) under identical acquisition settings across all experimental conditions. Cell‐associated fluorescence was quantified using ImageJ software and normalized to the number of nuclei.

For immunofluorescence analysis, AGS reporter cells were seeded onto glass coverslips and cultured for 24 h. Cells were pre‐incubated for 1 h with either a neutralizing anti‐TLR2 antibody (2 µg/mL) or the corresponding isotype control antibody (IgA), then stimulated with WT OMVs (10 µg/mL) for 5 h at 37°C. Cells were washed twice with PBS, fixed with 4% paraformaldehyde for 15 min at room temperature, washed three additional times with PBS, and blocked with PBS containing 4% bovine serum albumin (BSA). Samples were incubated overnight at 4°C with rabbit anti‐Golgin‐97 antibody (Sigma‐Aldrich ZRB1346) and mouse anti‐*H. pylori* OMP antibody (1:500 each). After washing, cells were incubated for 1 h at room temperature with goat anti‐mouse Alexa Fluor 488 (A10680) and donkey anti‐rabbit Alexa Fluor 594 (A11035) secondary antibodies (both 1:500; Thermo Fisher Scientific). Nuclei were counterstained with DAPI, coverslips were mounted using Fluoromount‐G, and fluorescence images were acquired using an Axioscope 5 epifluorescence microscope (Zeiss).

### 2.12 Isolation of Nuclear Protein Fractions

Nuclear and cytoplasmic protein fractions were isolated using a differential lysis protocol, a standard method for sequentially separating cellular compartments while maintaining protein integrity (Valenzuela et al. 2014). Initially, harvested cells were washed once with phosphate‐buffered saline (PBS, pH 7.2). The cell pellet was then gently resuspended in a hypotonic lysis buffer (Buffer A) to release the cytoplasmic contents. Buffer A consisted of 10 mM Hepes‐KOH (pH 7.9), 2 mM MgCl_2_, 0.1 mM EDTA, 10 mM KCl, 0.5% Nonidet P‐40 (NP40), 1 mM Dithiothreitol (DTT) and 0.5 mM Phenylmethylsulfonyl Fluoride (PMSF). Lysis was performed by incubating the cell suspension on ice for 10 min. Following incubation, the lysates were centrifuged at 13,000 × *g* for 1 min at 4°C. The resulting supernatant, containing the cytoplasmic fraction, was carefully collected and stored. The remaining pellet, which comprised the intact nuclei, was subsequently resuspended in a hypertonic extraction buffer (Buffer B) to facilitate the release of nuclear proteins. Buffer B was composed of 50 mM Hepes‐KOH (pH 7.9), 2 mM MgCl_2_, 0.1 mM EDTA, 400 mM NaCl, 50 mM KCl, 10% glycerol, 1 mM DTT and 0.5 mM PMSF. Nuclear protein extraction was achieved by allowing the nuclei to swell during a 30‐min incubation period on ice. The mixture was then centrifuged at 13,000 × *g* for 15 min at 4°C. The supernatant, representing the nuclear fraction, was recovered. Both the cytoplasmic and nuclear fractions were aliquoted and stored at −80°C for downstream analysis, typically involving immunoblotting.

### 2.13 Use of Artificial Intelligence–Assisted Tools

During the preparation of this manuscript, the authors used ChatGPT (OpenAI) to assist with language editing, improve clarity, and refine the wording of the Discussion and Conclusions. The authors determined the scientific interpretation of the results and the conclusions derived from the study. AI tools were not used to generate experimental data or perform data analysis. The authors critically reviewed and verified all AI‐assisted content against the experimental results and relevant literature, and take full responsibility for the integrity and final content of the manuscript.

### 2.14 Statistical Analysis of Data

All data are expressed as the means ± standard deviation of at least three independent experiments. Data were processed using GraphPad Prism v. 10.5.0 (774) (GraphPad Software, San Diego, CA, USA, www.graphpad.com).

## 3 Results

### 3.1 Assessment of NF‐κB Transcriptional Activity in Response to *H. pylori* OMVs and TLR2 Agonists

To assess NF‐κB transcriptional activation in response to *H. pylori* OMVs, stable gastric reporter cell lines were generated by hygromycin selection following transfection with the pNL3.2.NF‐κB‐RE plasmid, which drives NanoLuc expression under NF‐κB–responsive elements. The experimental setup is schematically illustrated in Figure 1A, showing cell stimulation in a 96‐well plate followed by luminescence measurement. AGS–NF‐κB RE and GES‐1–NF‐κB RE cells were exposed to OMVs isolated from *H. pylori* strains 60190 or 26695. As shown in Figure 1B,C, no NF‐κB activation was detected in either reporter line at any concentration tested (5–100 µg/mL) over 24 h, whereas TNF (20 ng/mL) robustly induced reporter activity, confirming assay functionality.

**FIGURE 1:**
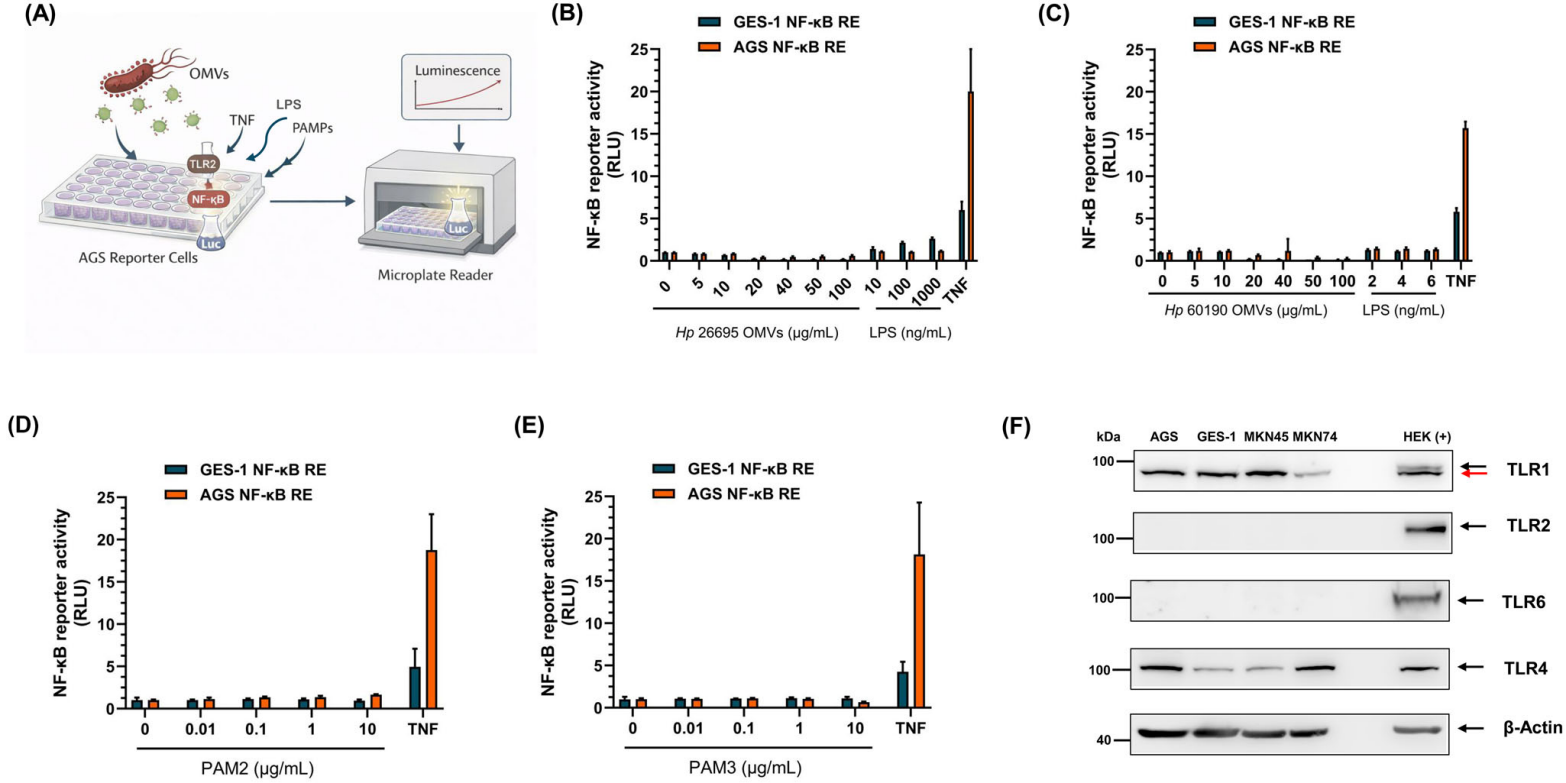
Lack of functional TLR2 heterodimers and NF‐κB responsiveness in gastric epithelial cells. (A) Schematic representation of the experimental workflow used to assess NF‐κB–dependent luminescent reporter activity in gastric epithelial cells, including stimulation in 96‐well plates followed by luminescence readout. (B, C) AGS–NF‐κB RE and GES‐1–NF‐κB RE reporter cell pools were treated for 24 h with increasing concentrations (5–100 µg/mL) of outer membrane vesicles (OMVs) isolated from *H. pylori* strains 26695 or 60190. Tumour necrosis factor (TNF; 20 ng/mL, 24 h) was included as a positive control. NF‐κB activation was quantified by luciferase assay and expressed as relative light units (RLU) relative to unstimulated control cells. (D, E) AGS–NF‐κB RE and GES‐1–NF‐κB RE reporter cells were stimulated with the synthetic TLR2 agonists Pam2CSK4 (PAM2) and Pam3CSK4 (PAM3) (0.01–10 µg/mL), which selectively activate TLR2/6 and TLR2/1 heterodimers, respectively. NF‐κB activation was quantified by luciferase assay and expressed as RLU relative to unstimulated control cells. (F) Western blot analysis of TLR2, TLR1, TLR6 and TLR4 expression in a panel of gastric epithelial cell lines. Protein lysates from HEK293 cells transiently transfected with pDuo‐TLR2/1 or pDuo‐TLR2/6 plasmids were included as positive controls. The lower–molecular–weight TLR1‐reactive species, consistent with a non‐glycosylated form, is indicated by a red arrow. A representative blot is shown. Data are presented as mean ± SD (*n* = 3).

Cells were next stimulated with the synthetic TLR2 agonists Pam2CSK4 (PAM2; Figure 1D) and Pam3CSK4 (PAM3; Figure 1E) (0.01–10 µg/mL), which activate TLR2/6 and TLR2/1 heterodimers, respectively. Neither AGS nor GES‐1 reporter cells responded to these ligands, indicating a lack of functional TLR2 heterodimers in these gastric epithelial models. Because OMVs contain lipopolysaccharide (LPS), *E. coli* O55:B5 LPS was also evaluated as a control for canonical TLR4 signalling. Even at high concentrations (up to 1 µg/mL), LPS induced only modest NF‐κB activation (Figure 1B,C), indicating that the gastric epithelial cell models used in this study display a weak NF‐κB response to LPS.

Given the lack of NF‐κB activation in response to OMVs, synthetic TLR2 agonists, and LPS, we next examined whether this unresponsiveness reflected deficient receptor expression. Basal protein levels of TLR2, TLR1, TLR6 and TLR4 were assessed by Western blot across a panel of gastric epithelial cell lines (Figure 1F). TLR2 and TLR6 were undetectable or expressed at extremely low levels. A lower‐molecular‐weight band reactive with the anti‐TLR1 antibody was consistently detected in gastric cell lysates; however, it migrated below the molecular weight of full‐length TLR1 ectopically expressed in HEK293 cells. Because its identity was not further investigated, this signal may correspond to a nonspecific band, an immature non‐glycosylated isoform, or a truncated receptor variant, as previously described for other members of the TLR family (Hart and Tapping 2012).

With respect to TLR4, a faint band of approximately 100 kDa corresponding to the immature unglycosylated isoform was detected, whereas the mature ∼130 kDa glycosylated form required for plasma membrane trafficking and canonical LPS sensing (Ohnishi et al. 2003) was absent. This expression pattern is consistent with the weak NF‐κB activation observed following LPS stimulation.

Taken together, these results indicate that the gastric epithelial cell models evaluated in this study lack detectable expression of functional TLR2 heterodimers and do not express the fully glycosylated, membrane‐localized TLR4 isoform required for canonical LPS recognition. Although the identity of the lower‐molecular‐weight TLR1‐reactive band remains unresolved, the absence of functional responses to synthetic TLR2 agonists supports the conclusion that functional TLR2 heterodimers are not present under these experimental conditions. Collectively, these findings provide a mechanistic explanation for the inability of the gastric epithelial cell models used in this study to activate NF‐κB in response to *H. pylori* OMVs, synthetic TLR2 agonists, or *E. coli* LPS.

### 3.2 Evaluation of NF‐κB Reporter Activity in Response to TLR2 Agonists in Gastric Epithelial Cells Constitutively Expressing TLR2/1 and TLR2/6 Heterodimers

TLR2 is detectable in normal gastric epithelium and is frequently overexpressed in gastric cancer (West et al. 2017). However, immunodetection analyses revealed no detectable expression of functional TLR2 heterodimers in commonly used gastric epithelial cell lines, consistent with their inability to mount NF‐κB responses to synthetic TLR2 agonists. This limitation likely obscures potential OMV‐induced signalling mediated through TLR2 in these models. To overcome this constraint, we generated stable AGS–NF‐κB RE and GES‐1–NF‐κB RE reporter cell lines constitutively expressing either the TLR2/1 or TLR2/6 heterodimers using the pDuo‐TLR2/1 and pDuo‐TLR2/6 expression plasmids.

As shown in Figure 2, expression of the TLR2/6 heterodimer in GES‐1–NF‐κB RE (Figure 2B) and AGS–NF‐κB RE (Figure 2E) reporter cells conferred robust and reproducible NF‐κB activation in response to TLR2 agonist stimulation across three independently selected clones. For this initial screening, relatively high agonist concentrations were used due to the limited responsiveness of parental cells. This likely explains the activation of both TLR2/1‐ and TLR2/6‐expressing reporter cells by Pam3CSK4 (PAM3), a TLR2/1‐selective agonist. Because this initial screening was designed to identify responsive clones rather than to assess agonist selectivity, the specificity of the selected reporter cell lines was subsequently evaluated by dose‐response experiments using lower agonist concentrations (Figure 3A–D), which confirmed the expected preferential activation profiles of the corresponding TLR2 heterodimers. In contrast, these reporter cells showed no or only weak responses to *E. coli* LPS (1 µg/mL), supporting the conclusion that endogenous TLR4 expression is insufficient to elicit robust NF‐κB activation.

**FIGURE 2:**
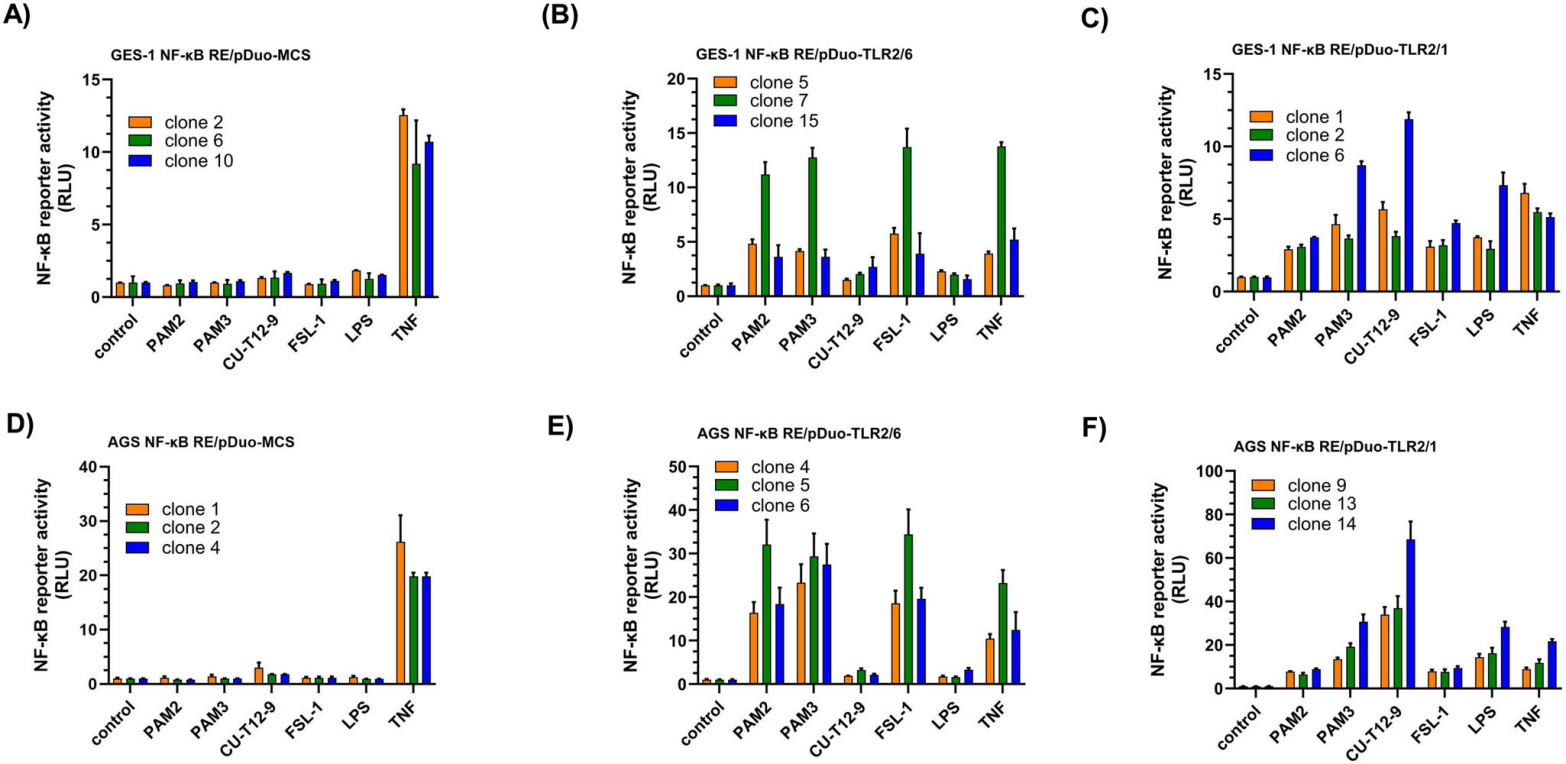
Functional reconstitution of TLR2 heterodimers enables NF‐κB activation by synthetic TLR2 agonists in gastric epithelial cells. (A, D) GES‐1–NF‐κB RE (A) and AGS–NF‐κB RE (D) reporter cells transfected with the empty control plasmid (pDuo‐MCS) were stimulated with synthetic TLR2 agonists (Pam2CSK4, PAM2, 10 µg/mL; Pam3CSK4, PAM3, 10 µg/mL; CU‐T12‐9, 10 µM; or FSL‐1, 10 µg/mL) or with *Escherichia coli* LPS (1 µg/mL) and analyzed for NF‐κB–dependent reporter activity. Tumour necrosis factor (TNF; 20 ng/mL) was included as a positive control. (B, E) GES‐1–NF‐κB RE (B) and AGS–NF‐κB RE (E) reporter cells constitutively expressing the TLR2/6 heterodimer were stimulated with the indicated TLR2 agonists, *E. coli* LPS, or TNF. (C, F) GES‐1–NF‐κB RE (C) and AGS–NF‐κB RE (F) reporter cells constitutively expressing the TLR2/1 heterodimer were stimulated with the indicated agonists under the same conditions. NF‐κB activation was quantified by luciferase assay and expressed as relative light units (RLU) relative to unstimulated control cells. Bars represent the mean ± SD (*n* = 3).

**FIGURE 3:**
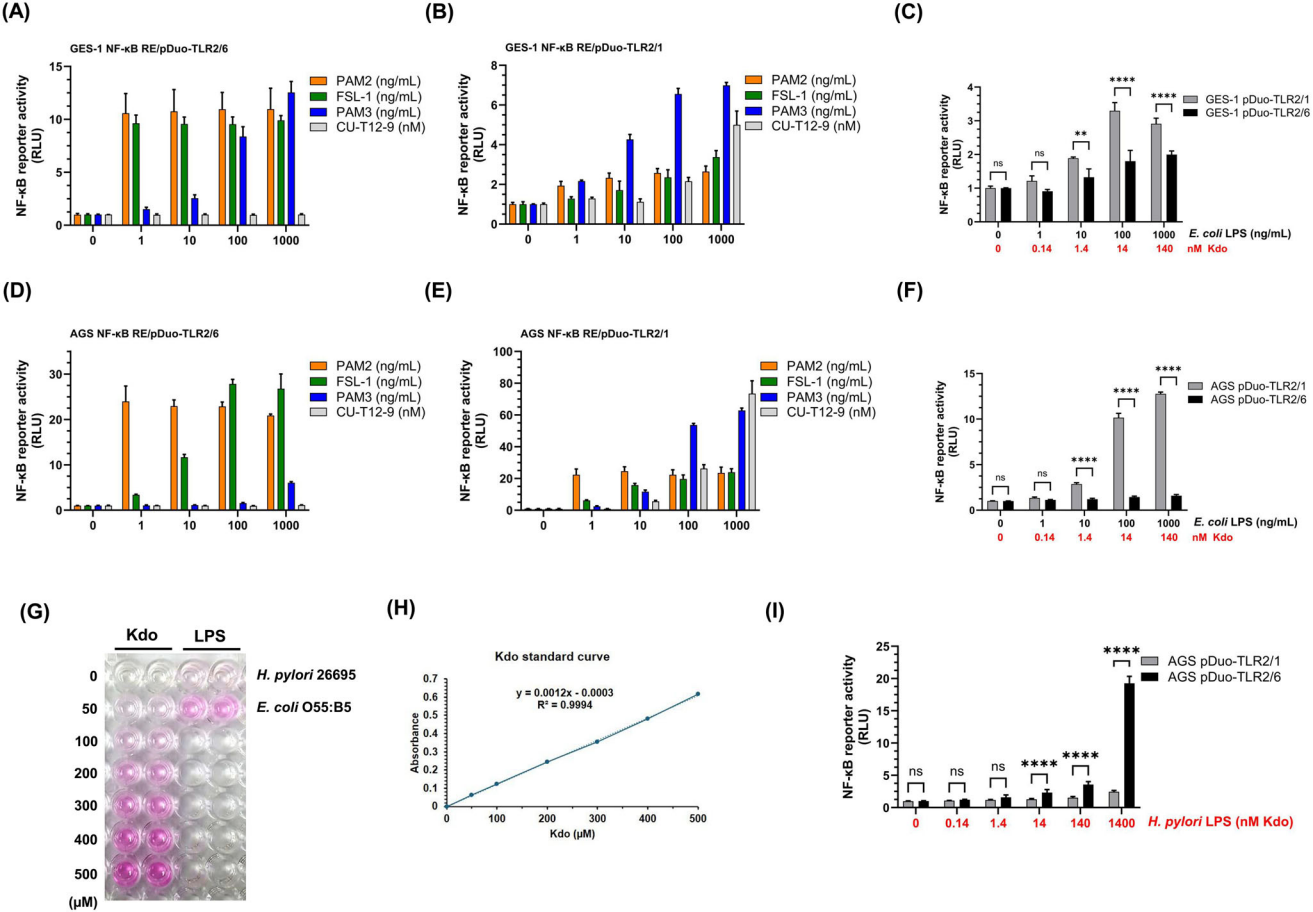
Functional validation of TLR2/1‐ and TLR2/6‐specific NF‐κB reporter cells using heterodimer‐selective agonists and purified LPS. (A, B, D, E) GES‐1 (A, B) and AGS (D, E) NF‐κB reporter cells stably expressing either the TLR2/6 or TLR2/1 heterodimer were stimulated for 5 h with the TLR2/6‐selective agonists FSL‐1 or Pam2CSK4 (Pam2), or with the TLR2/1‐selective agonists CU‐T12‐9 or Pam3CSK4 (Pam3), over the indicated concentration ranges (1–1,000 ng/mL for peptide agonists and 1–1,000 nM for CU‐T12‐9). (C, F) GES‐1 (C) and AGS (F) reporter cells expressing either TLR2/1 or TLR2/6 were stimulated for 5 h with increasing concentrations of purified *E. coli* LPS (1–1,000 ng/mL; 0.14–140 nM Kdo equivalents). (G) Representative colorimetric Kdo assay used for LPS quantification. (H) Standard curve generated with commercially available 2‐keto‐3‐deoxyoctonate (Kdo) and used for normalization of purified LPS preparations. (I) AGS NF‐κB reporter cells expressing either the TLR2/1 or TLR2/6 heterodimer were stimulated for 5 h with purified *H. pylori* strain 26695 LPS normalized according to Kdo content (0.14–1,400 nM Kdo equivalents). NF‐κB‐dependent luminescent reporter activity was measured after stimulation and expressed as relative light units (RLUs) normalized to untreated controls. Bars represent the mean ± SD of three independent experiments. Statistical significance is indicated as *p < 0.05, **p < 0.01, ***p < 0.001, ****p < 0.0001.

Conversely, expression of the TLR2/1 heterodimer in GES‐1 (Figure 2C) and AGS (Figure 2F) reporter cells conferred responsiveness to the TLR2/1 agonists CU‐T12‐9 and PAM3, whereas it elicited weaker responses to TLR2/6‐selective agonists. This response pattern was more pronounced in AGS‐derived clones, which, overall, exhibited stronger NF‐κB activation in response to TLR2/1 agonists than GES‐1 clones. Notably, expression of the TLR2/1 heterodimer also enabled reporter cells to respond to *E. coli* LPS (Figure 2C,F), consistent with previously reported cross‐talk between TLR2‐ and TLR4‐dependent signalling pathways (Kirschning et al. 1998).

In contrast, reporter clones derived from GES‐1 (Figure 2A) or AGS (Figure 2D) cells transfected with the empty control plasmid (pDuo‐MCS) failed to respond to any of the tested TLR2 agonists or to *E. coli* LPS, while retaining responsiveness to TNF stimulation. This confirms that NF‐κB activation in the engineered reporter cells is strictly dependent on heterodimer expression. Although individual clones exhibited quantitative differences in agonist responsiveness, their overall activation profiles were consistent. Clones displaying comparable responses to TNF were therefore selected for subsequent experiments.

### 3.3 Functional Discrimination of TLR2 Heterodimers in Gastric Epithelial NF‐κB Reporter Cells

As indicated in the previous section, saturating agonist concentrations were initially used to identify and select TLR2/NF‐κB reporter clones. We next evaluated the agonist selectivity of these reporter cell lines using dose‐response analyses performed with concentrations commonly employed in comparable studies. For this purpose, FSL‐1 and Pam2CSK4 (PAM2) were used as TLR2/6‐selective agonists, whereas CU‐T12‐9 and Pam3CSK4 (PAM3) were used as TLR2/1‐selective agonists.

As shown in Figure 3, GES‐1 (Figure 3A) and AGS (Figure 3D) NF‐κB reporter cells expressing the TLR2/6 heterodimer responded robustly to FSL‐1 and PAM2 at 10 ng/mL, while showing no detectable activation in response to CU‐T12‐9 (10 µM) or PAM3 (10 ng/mL). Notably, AGS NF‐κB RE/pDuo‐TLR2/6 reporter cells retained this discriminatory capacity even when challenged with higher agonist concentrations. In contrast, gastric reporter cells expressing the TLR2/1 heterodimer (GES‐1, Figure 3B; AGS, Figure 3E) responded strongly to both PAM3 and CU‐T12‐9 but also exhibited partial responsiveness to the TLR2/6‐selective agonists, indicating lower ligand selectivity than TLR2/6‐expressing cells.

We next evaluated NF‐κB activation in response to purified *E. coli* LPS. As shown in Figure 3C,F, *E. coli* LPS induced NF‐κB activation predominantly in reporter cells expressing the TLR2/1 heterodimer, whereas TLR2/6‐expressing cells remained unresponsive or displayed only minimal activation. To determine whether purified *H. pylori* LPS exhibited a similar activation profile, LPS preparations were first normalized according to their Kdo content using a standard curve generated with commercially available 2‐keto‐3‐deoxyoctonate (Kdo) (Figure 3G,H). Purified *H. pylori* LPS was then tested at a Kdo‐equivalent concentration tenfold higher than that used for *E. coli* LPS to maximize the likelihood of detecting TLR2‐dependent signalling. Under these conditions, *H. pylori* LPS induced a robust NF‐κB response in TLR2/6‐reconstituted cells but failed to activate TLR2/1‐reconstituted cells (Figure 3I). In contrast, *E. coli* LPS elicited a strong response in TLR2/1‐reconstituted cells, although only at concentrations substantially higher than those typically used to stimulate the canonical LPS receptor TLR4 (Cochet and Peri 2017). These findings confirm that purified *H. pylori* LPS is capable of activating TLR2‐dependent signalling in our experimental system while revealing a heterodimer activation profile distinct from that of purified *E. coli* LPS. Importantly, the selected reporter clones constitute a robust and functionally validated platform for dissecting TLR2 heterodimer‐specific NF‐κB signalling and were therefore used in all subsequent experiments evaluating the effects of *H. pylori*‐derived OMVs.

### 3.4 Ectopic Expression of TLR2/1 and TLR2/6 Heterodimers Confers Responsiveness to *H. pylori* OMVs in Gastric NF‐κB Reporter Cells

Reporter AGS cells were previously identified as the most suitable model for analyzing the effects of *H. pylori* OMVs, compared with GES‐1 cells, owing to their robust NF‐κB responsiveness and their ability to discriminate among different ligands. Accordingly, AGS NF‐κB reporter cells were used to assess OMV‐induced signalling in subsequent experiments. As shown in Figure 4, AGS reporter cells expressing either the TLR2/1 or TLR2/6 heterodimer exhibited a dose‐dependent increase in NF‐κB reporter activity upon stimulation with OMVs (2.5–20 µg/mL) derived from *H. pylori* strain 26695 (Figure 4A) or strain 60190 (Figure 4B). In contrast, control cells transfected with the empty vector (AGS pDuo‐MCS) failed to respond to OMVs at any of the concentrations tested, indicating that heterodimer expression is required for OMV‐induced NF‐κB activation. To determine whether OMV‐induced signalling was directly dependent on TLR2 heterodimer engagement, reporter cells were pre‐incubated with neutralizing antibodies against TLR1, TLR2 or TLR6 before OMV stimulation. As shown in Figure 4C–F, pre‐treatment with a TLR2‐neutralizing antibody markedly reduced NF‐κB activation in response to OMVs derived from strain 26695 in both TLR2/6‐expressing cells (Figure 4C) and TLR2/1‐expressing cells (Figure 4D). A comparable inhibitory effect was observed following TLR2 neutralization in response to OMVs derived from strain 60190, in both TLR2/6 (Figure 4E) and TLR2/1 (Figure 4F) reporter cells. In contrast, neutralization of TLR1 or TLR6 resulted in a significant but partial reduction of OMV‐induced NF‐κB activation for both *H. pylori* strains, consistent with the contribution of both heterodimeric configurations to OMV sensing.

**FIGURE 4:**
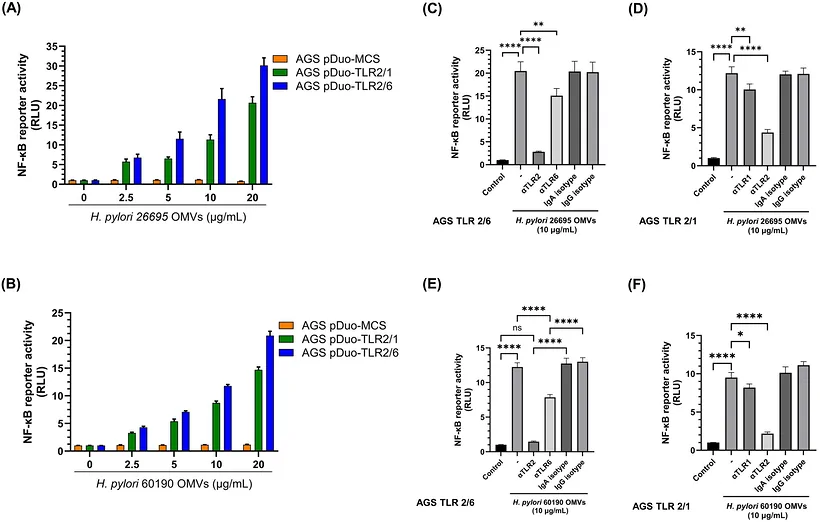
*Helicobacter pylori* outer membrane vesicles (OMVs) activate NF‐κB signalling through TLR2‐containing heterodimers. AGS NF‐κB reporter cells stably expressing the TLR2/1 or TLR2/6 heterodimer, or transfected with the empty vector control (pDuo‐MCS), were stimulated for 5 h with increasing concentrations (2.5–20 µg/mL) of OMVs isolated from *H. pylori* strains 26695 (A) or 60190 (B). NF‐κB–dependent luminescent reporter activity was measured after stimulation and expressed as relative light units (RLUs) relative to untreated controls. For receptor neutralization assays, AGS NF‐κB reporter cells expressing TLR2/1 or TLR2/6 were preincubated for 1 h with neutralizing antibodies against TLR1, TLR2 or TLR6 before stimulation with OMVs derived from *H. pylori* strain 26695 (C, D) or strain 60190 (E, F) for 5 h. NF‐κB–dependent reporter activity was quantified by luminescence and expressed relative to untreated controls as RLU. Bars represent the mean ± SD (*n* = 3). Statistical significance is indicated as *p < 0.05, **p < 0.01, ***p < 0.0001, and ns, not significant.

Together, these data demonstrate that *H. pylori* OMVs induce TLR2‐dependent NF‐κB activation in AGS gastric epithelial reporter cells through both TLR2/1 and TLR2/6 heterodimers.

### 3.5 Role of *H. pylori* Urease in OMV‐Mediated Activation of the TLR2/NF‐κB Pathway

Because previous studies have implicated *H. pylori* urease in TLR2‐dependent signalling (Kaparakis et al. 2010, Fu and Lai 2022), we investigated whether urease contributes to the immunostimulatory activity of *H. pylori* OMVs derived from the *H. pylori* 60190 wild‐type strain and its isogenic urease‐deficient mutant (Δ*ure*). As shown in Figure 5A, SDS–PAGE analysis confirmed the presence of the urease subunits UreA (∼26 kDa) and UreB (∼62 kDa) in OMVs derived from the wild‐type strain, whereas both proteins were absent from OMVs isolated from the Δ*ure* mutant. Consistently, urease activity, assessed by phenol red colour change, was detected in protein extracts from wild‐type OMVs but not from Δ*ure* OMVs (Figure 5A, lower panel). Nanoparticle tracking analysis revealed that OMVs derived from the wild‐type and Δ*ure* strains displayed comparable size distributions, with a predominant vesicle population ranging from approximately 100 to 150 nm, indicating that urease deletion did not significantly affect OMV size or vesicle production (Figure 5B). We next assessed the ability of wild‐type and Δ*ure* OMVs to activate NF‐κB signalling in AGS NF‐κB reporter cells stably transfected with the empty vector control (pDuo‐MCS) or expression constructs encoding the TLR2/1 or TLR2/6 heterodimers. Cells were stimulated with OMVs from either strain at concentrations of 10 or 20 µg/mL. At 10 µg/mL, OMVs derived from the wild‐type and Δ*ure* strains induced comparable levels of NF‐κB activation in cells expressing either TLR2/1 or TLR2/6, with no statistically significant differences between conditions (Figure 5C). In contrast, at 20 µg/mL, OMVs from the Δ*ure* strain elicited a modest but reproducible reduction in NF‐κB activation compared with wild‐type OMVs in cells expressing both TLR2/1 and TLR2/6 heterodimers (Figure 5C). As expected, cells transfected with the empty vector did not exhibit NF‐κB activation under any of the conditions tested. To validate these findings, OMVs derived from the *H. pylori* 26695 strain background were also examined. Consistent with the results obtained using strain 60190, differences between wild‐type and Δ*ure* OMVs were minimal at lower concentrations. In contrast, at 20 µg/mL, a reduced NF‐κB response was again observed in TLR2 heterodimer–expressing cells stimulated with Δ*ure* OMVs (Figure 5D).

**FIGURE 5:**
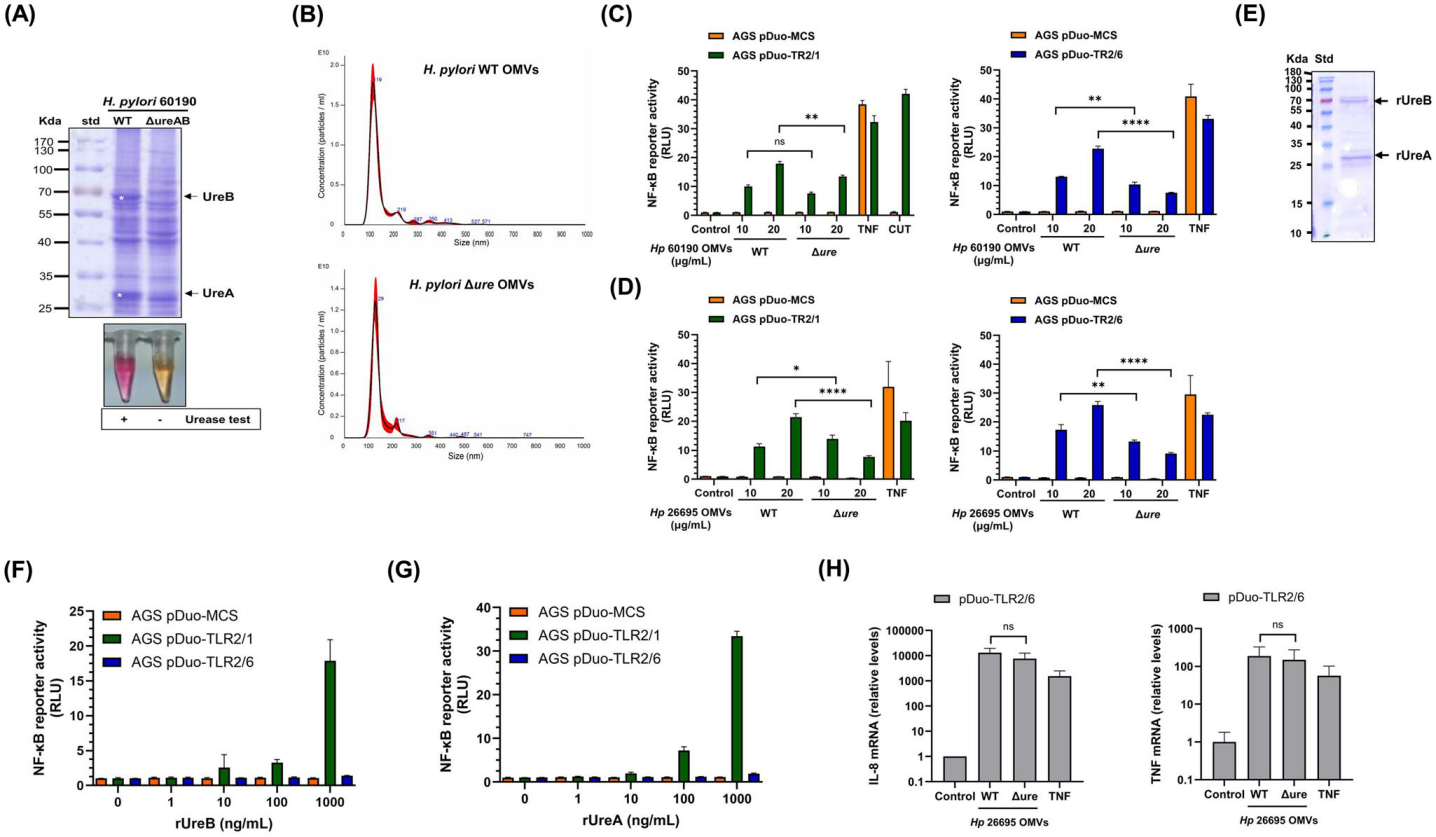
Contribution of *Helicobacter pylori* urease to OMV‐mediated TLR2–NF‐κB activation. (A) SDS–PAGE analysis of OMVs derived from the *H. pylori* 60190 wild‐type (WT) strain and its isogenic urease‐deficient mutant (Δ*ure*), showing the presence of the urease subunits UreA (∼26 kDa) and UreB (∼62 kDa) in WT OMVs and their absence in Δ*ure* OMVs. Urease activity was assessed by phenol red colour change. (B) Nanoparticle tracking analysis (NTA) showing comparable size distributions of WT and Δ*ure* OMVs, with a predominant vesicle population between approximately 100 and 150 nm. (C) NF‐κB activation in AGS reporter cells expressing TLR2/1 or TLR2/6 heterodimers following stimulation with OMVs derived from the *H. pylori* 60190 WT or Δ*ure* strains at concentrations of 10 or 20 µg/mL. Cells transfected with the empty vector (pDuo‐MCS) served as negative controls. (D) NF‐κB activation in AGS reporter cells expressing TLR2 heterodimers following stimulation with OMVs derived from the *H. pylori* 26695 WT or Δ*ure* strains, showing reduced signalling in response to Δ*ure* OMVs at higher concentrations. (E) SDS–PAGE analysis of recombinant UreA and UreB proteins expressed in *Escherichia coli* BL21(DE3) and purified by Ni^2^^+^‐NTA affinity chromatography, followed by Coomassie blue staining. (F, G) NF‐κB activation in AGS reporter cells expressing TLR2/1, TLR2/6 or the empty vector following stimulation with increasing concentrations of recombinant UreA (F) or UreB (G), showing preferential activation through TLR2/1. (H) Relative mRNA expression levels of IL‐8 and TNF in AGS cells expressing TLR2/6 following stimulation with WT or Δ*ure* OMVs (10 µg/mL) from *H. pylori* 26695, showing comparable induction of proinflammatory gene expression. NF‐κB–dependent luminescent reporter activity was measured after stimulation and expressed relative to untreated controls as relative light units (RLU). Gene expression data were normalized to housekeeping genes and expressed relative to untreated controls. Bars represent the mean ± SD (*n* = 3). Statistical significance is indicated as *p < 0.05, **p < 0.01, ****p < 0.0001; and ns, non‐significant.

To further dissect the contribution of individual urease subunits, recombinant UreA and UreB proteins were expressed in *E. coli* BL21(DE3), purified by Ni^2^^+^‐NTA affinity chromatography, and verified by SDS–PAGE followed by Coomassie blue staining (Figure 5E). As shown in Figure 5F,G, both recombinant proteins induced NF‐κB activation in a concentration‐dependent manner in AGS cells expressing TLR2/1, with UreA eliciting a stronger response than UreB. Notably, robust NF‐κB activation was observed only at higher protein concentrations, whereas lower concentrations induced weak or negligible responses. In contrast, neither recombinant UreA nor UreB induced substantial NF‐κB activation in cells expressing TLR2/6 or in cells transfected with the empty vector control.

Consistent with the reporter assays, OMVs derived from the Δ*ure* mutant induced IL‐8 and TNF mRNA expression to levels comparable to those elicited by wild‐type OMVs in AGS cells expressing TLR2/6 (Figure 5H). No statistically significant differences were observed between WT and Δ*ure* OMVs, indicating that urease is dispensable for OMV‐driven proinflammatory gene induction at the transcriptional level.

Taken together, these results indicate that urease contributes to OMV‐induced NF‐κB activation in gastric epithelial cells in a concentration‐dependent manner. While its impact is limited at lower OMV or recombinant protein concentrations, a modest but reproducible effect becomes evident at higher doses, supporting a modulatory rather than dominant role for urease in OMV‐mediated TLR2 signalling. Importantly, the preservation of robust inflammatory signalling in Δ*ure* OMVs indicates that other OMV‐associated components play a more prominent role in TLR2‐dependent activation. We therefore next investigated alternative OMV‐associated virulence factors driving this response.

### 3.6 NapA Is a Major OMV‐Associated Determinant of TLR2‐Dependent NF‐κB Activation

Based on our previous observations indicating that urease contributes to OMV‐induced TLR2 activation only at higher concentrations and plays a modulatory rather than dominant role, we next sought to identify the major OMV‐associated determinant contributing to TLR2‐mediated signalling in gastric epithelial cells. To this end, OMVs derived from the *H. pylori* 26695 wild‐type strain and from isogenic mutants lacking selected virulence‐associated factors were analyzed at a fixed concentration of 10 µg/mL, a condition that consistently elicited robust NF‐κB activation in our reporter assays.

Under these conditions, wild‐type OMVs induced strong NF‐κB reporter activation in AGS cells expressing either TLR2/1 or TLR2/6 heterodimers, with a consistently higher response observed in TLR2/6‐expressing cells. In contrast, no activation was detected in cells transfected with the empty vector control (Figure 6A). Notably, deletion of *napA* resulted in the most pronounced reduction of NF‐κB activity in cells expressing either TLR2/1 or TLR2/6. In contrast, OMVs derived from the Δ*ure* mutant and from mutants lacking other virulence‐associated factors, including O‐antigen–deficient LPS (Δhp0826 and Δhp1039), Δ*vacA*, and Δ*cagA*, retained substantial NF‐κB–stimulating activity, particularly in cells expressing TLR2/1 (Figure 6A). Notably, OMVs derived from the Δ*vacA* mutant showed a significant reduction in NF‐κB activation, specifically in TLR2/1‐expressing cells. Collectively, these results indicate that although multiple OMV‐associated components contribute to TLR2‐mediated signalling, NapA represents the major OMV‐associated determinant of OMV‐induced TLR2 activation under these conditions.

**FIGURE 6:**
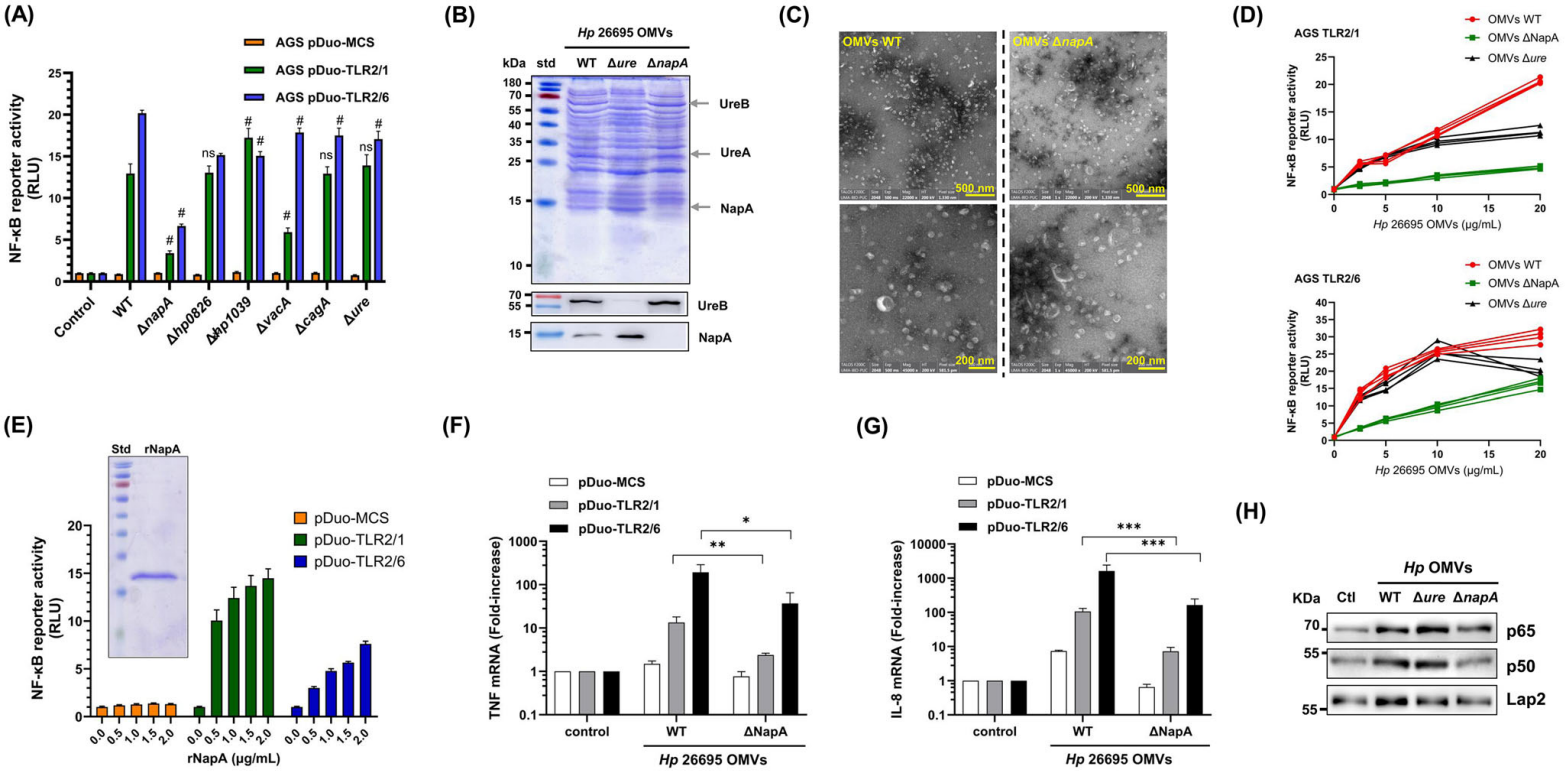
NapA is the major OMV‐associated determinant driving TLR2‐dependent NF‐κB activation and proinflammatory responses in gastric epithelial cells. (A) NF‐κB activation in AGS reporter cells stably expressing TLR2/1 or TLR2/6 heterodimers following stimulation for 5 h with OMVs derived from the *H. pylori* 26695 wild‐type (WT) strain or from isogenic mutants lacking selected virulence‐associated factors, including urease (Δ*ure*), NapA (Δ*napA*), O‐antigen–deficient LPS (Δ*hp0826* and Δ*hp1039*), VacA (Δ*vacA*), or CagA (Δ*cagA*). OMVs were used at a fixed concentration of 10 µg/mL. Cells transfected with the empty vector (pDuo‐MCS) served as negative controls. (B) SDS–PAGE analysis followed by Coomassie blue staining of OMVs derived from the wild‐type, Δ*ure* and Δ*napA* strains showing broadly comparable protein profiles among vesicle preparations. Lower panels: immunoblot analysis using anti‐UreB and anti‐NapA antibodies confirmed the selective absence of UreB and NapA in OMVs derived from the corresponding mutant strains. Increased NapA abundance was consistently observed in Δ*ure* OMVs compared with WT OMVs. NapA immunoreactivity migrated at approximately 14–15 kDa under the electrophoretic conditions used. (C) Representative TEM images of wild‐type and Δ*napA* OMVs are shown at low and higher magnification. Scale bars: 500 nm and 200 nm, as indicated. TEM analysis showed vesicle‐like structures with comparable morphology between wild‐type and Δ*napA* preparations. (D) Dose–response analysis of NF‐κB activation in AGS reporter cells expressing TLR2/1 (upper panel) or TLR2/6 (lower panel) following stimulation with increasing concentrations of OMVs derived from WT, Δ*napA* or Δ*ure* strains. (E) NF‐κB activation in AGS reporter cells expressing TLR2/1 or TLR2/6 following stimulation with increasing concentrations of recombinant NapA (rNapA). Cells transfected with the empty vector served as controls. The inset shows an SDS–PAGE analysis of purified rNapA, stained with Coomassie Brilliant Blue. (F, G) Relative mRNA expression levels of TNF (F) and IL‐8 (G) in AGS cells expressing TLR2/1 or TLR2/6 following stimulation with WT or Δ*napA* OMVs (10 µg/mL), as determined by RT–qPCR. (H) Immunoblot analysis of NF‐κB nuclear translocation in TLR2/6‐expressing AGS cells following stimulation with WT, Δ*ure* or Δ*napA* OMVs. A representative blot is shown. Bars represent the mean ± SD (*n* = 3). Statistical significance is indicated as ns (not significant), *p < 0.05, **p < 0.01, ***p < 0.001 and ^#^ *p*<0.0001.

To investigate whether the effect observed for the Δ*ure* mutant at higher OMV concentrations was associated with changes in vesicular NapA abundance, the protein composition and morphology of OMVs were further analyzed. As shown in Figure 6B, SDS–PAGE analysis followed by Coomassie blue staining revealed broadly comparable OMV protein profiles among strains. The identity and selective loss of UreB and NapA in mutant‐derived OMVs were further confirmed by immunoblotting with anti‐UreB and anti‐NapA antibodies, respectively (Figure 6B, lower panels). NapA immunoreactivity was detected in wild‐type and Δ*ure* OMVs but was absent in Δ*napA* OMVs, whereas UreB was absent only in OMVs derived from the Δure mutant, validating the molecular composition of the vesicle preparations. Although NapA has a predicted molecular weight of approximately 17 kDa, the anti‐NapA immunoreactive band consistently migrated at ∼14–15 kDa under our SDS–PAGE conditions. Transmission electron microscopy further showed that WT and Δ*napA* OMVs exhibited comparable vesicular morphology and size range, supporting that the observed functional differences were not associated with major alterations in vesicle integrity or morphology (Figure 6C). Notably, OMVs derived from the Δ*ure* mutant consistently exhibited increased levels of NapA compared with wild‐type OMVs. Despite this increased abundance, Δ*ure* OMVs did not exhibit enhanced TLR2‐dependent NF‐κB activation. These observations indicate that vesicular NapA abundance alone is insufficient to explain OMV‐induced TLR2 signalling. Instead, they suggest that the immunostimulatory activity of NapA depends on its presentation within the OMV context.

Dose–response analyses revealed that wild‐type OMVs induced concentration‐dependent NF‐κB activation in AGS cells expressing either TLR2/1 or TLR2/6 (Figure 6D). In contrast, OMVs derived from the Δ*napA* mutant exhibited a markedly attenuated NF‐κB response that remained consistently lower than that induced by WT OMVs throughout the concentration range tested. By comparison, Δ*ure* OMVs displayed activation profiles comparable to those of wild‐type OMVs at low and intermediate concentrations, further supporting the notion that urease is not a limiting factor for TLR2 activation under these experimental conditions (Figure 6D).

To further delineate the contribution of NapA to TLR2 engagement, we compared the stimulatory capacity of OMV‐associated NapA with that of purified recombinant NapA (rNapA). Although recombinant NapA induced dose‐dependent NF‐κB activation in both TLR2/1‐ and TLR2/6‐expressing reporter cells (Figure 6E), the effective concentrations required to elicit a significant response were substantially higher than those present within intact OMVs. Notably, stimulation with 2.5 µg/mL of wild‐type OMVs (Figure 6C)—which contain NapA as only a fraction of its total protein cargo—triggered a more robust NF‐κB response than saturating concentrations of soluble rNapA. This discrepancy indicates that the presentation of NapA within the OMV architecture markedly enhances its bioactivity, likely through multivalent receptor engagement or improved stability at the host cell surface. The purity of the recombinant NapA used in these assays was verified by SDS–PAGE followed by Coomassie blue staining (Figure 6E, inset).

Consistent with the NF‐κB reporter assays, stimulation with wild‐type OMVs at 10 µg/mL resulted in robust upregulation of IL‐8 and TNF mRNA expression in AGS cells expressing either TLR2/1 or TLR2/6 (Figure 6F,G, respectively). In contrast, stimulation with Δ*napA* OMVs led to a markedly attenuated cytokine response, with transcript levels induced at approximately one order of magnitude lower than those elicited by wild‐type OMVs. To assess whether NapA‐dependent OMV stimulation resulted in functional NF‐κB activation, we analyzed nuclear translocation of the NF‐κB subunits p65 and p50. Stimulation of TLR2/6‐expressing AGS cells with wild‐type and Δ*ure H. pylori* OMVs induced a clear accumulation of both p65 and p50 in nuclear fractions within 45 min, whereas OMVs derived from the Δ*napA* strain failed to efficiently promote NF‐κB nuclear translocation (Figure 6H).

Together, these results demonstrate that, although urease may modulate TLR2 signalling under specific high‐dose conditions, NapA is the principal OMV‐associated factor driving TLR2‐dependent NF‐κB activation and downstream proinflammatory gene expression in gastric epithelial cells.

### 3.7 OMV‐Associated NapA Promotes the TLR2‐Dependent Association of *H. pylori* OMVs With Gastric Epithelial Cells

Having established that NapA is a major OMV‐associated determinant of TLR2‐dependent NF‐κB activation and downstream pro‐inflammatory gene expression, we next asked whether TLR2 expression also influences the early physical association of *H. pylori* OMVs with gastric epithelial cells and whether NapA contributes to this process. To address this, OMV–cell interactions were analyzed by epifluorescence microscopy using AGS NF‐κB reporter cell lines expressing different TLR2 heterodimers. Consistent with a role for TLR2 in OMV recognition, OMVs derived from the *H. pylori* 26695 strain associated markedly less with mock‐transfected control (MCS) cells than with AGS cells expressing either the TLR2/1 or TLR2/6 heterodimers (Figure 7A). Quantitative analysis of OMV‐associated fluorescence confirmed a significant increase in OMV association in TLR2‐expressing cells relative to MCS controls (Figure 7B), indicating that TLR2 heterodimers facilitate OMV interaction with gastric epithelial cells.

**FIGURE 7:**
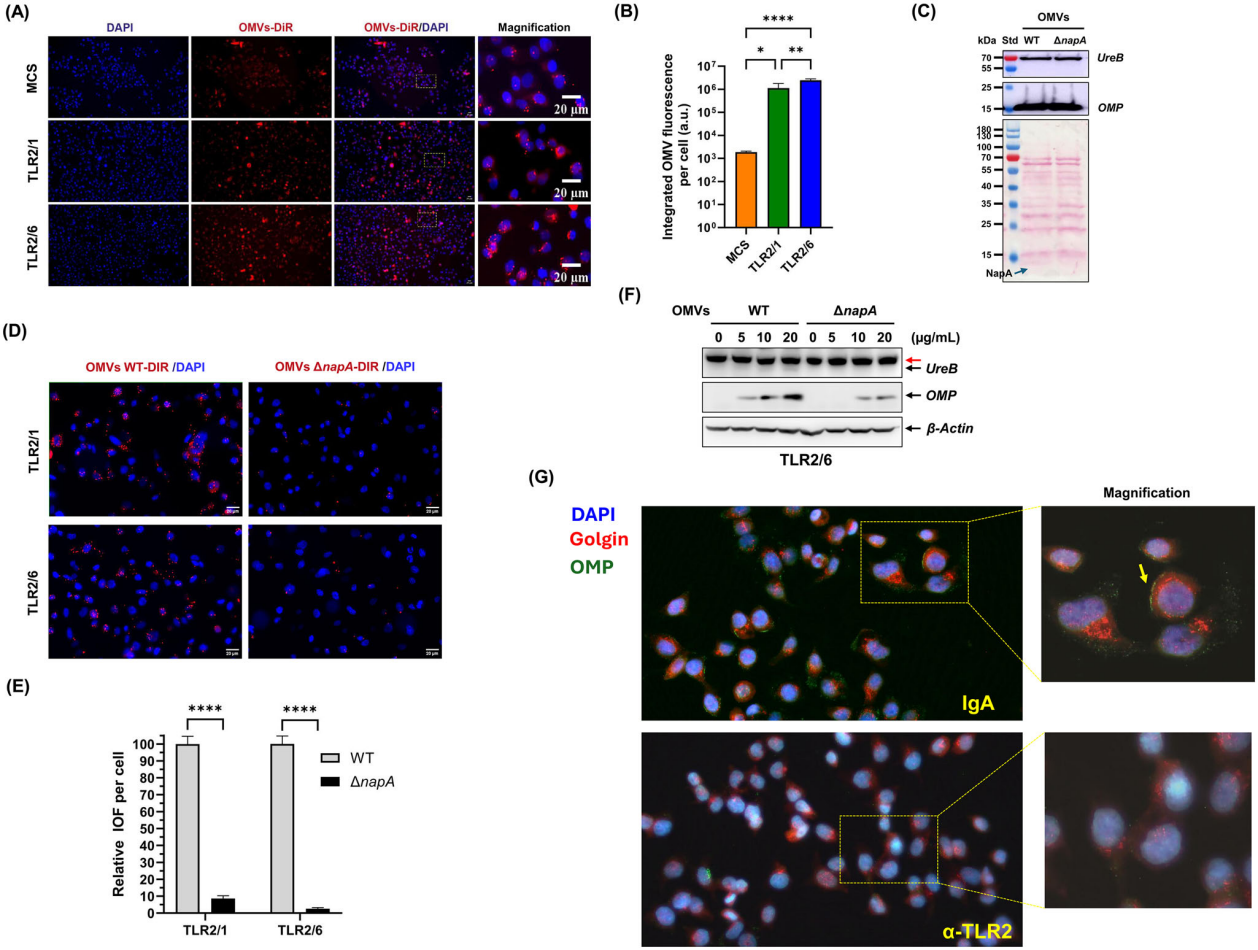
TLR2 expression and NapA promote the association of *H. pylori* 26695 OMVs with AGS reporter cells. (A) Fluorescence microscopy images show the interaction of DiR‐labeled *H. pylori* outer membrane vesicles (OMVs‐DiR, red) with AGS reporter cells expressing TLR2/1, TLR2/6 or mock control cells (MCS). Cell nuclei were counterstained with DAPI (blue). Representative merged images and higher‐magnification views are shown. Scale bars, as indicated. (B) Quantification of integrated OMV fluorescence per cell demonstrates increased association of OMVs with TLR2/1‐ and TLR2/6‐expressing cells compared with mock control cells. Data are presented as mean ± SD, and statistical significance is indicated (*p < 0.05, **p < 0.01 and ****p < 0.0001). (C) Ponceau S staining and immunoblot analysis of OMVs derived from wild‐type (WT) and Δ*napA* strains (20 µg) detected an outer membrane protein (OMP, ∼17 kDa) and the urease subunit UreB, indicating comparable protein loading across samples. (D) Representative fluorescence microscopy images comparing the association of wild‐type (WT) OMVs and OMVs derived from a Δ*napA* mutant strain with TLR2/1‐ and TLR2/6‐expressing cells are shown. Bar scale: 20 µm. (E) Quantitative analysis of relative integrated OMV fluorescence (IOF) per cell reveals a marked reduction in OMV association in the absence of NapA, indicating that NapA contributes to efficient OMV–TLR2 interaction. Data are expressed as mean ± SD (****p < 0.0001). (F) Immunoblot analysis of protein extracts from TLR2/6‐expressing AGS reporter cells treated for 5 h with wild‐type or Δ*napA* OMVs at 5, 10 and 20 µg/mL, detecting OMV‐associated proteins (OMP and UreB) and β‐actin as a cellular loading control. The red arrow indicates a band corresponding to a cellular protein that is nonspecifically recognized by the anti‐UreB antibody. Blots are representative of at least three independent experiments. (G) AGS‐TLR2/6 cells were pre‐incubated with an anti‐TLR2 neutralizing antibody or the corresponding isotype control (IgA) prior to stimulation with WT OMVs for 5 h. Cells were fixed and immunostained with an anti‐*H. pylori* OMP antibody (green), an anti‐Golgin antibody (red) and DAPI (blue). Representative immunofluorescence images show OMV‐associated fluorescence at the cell surface and as discrete puncta within the perinuclear region adjacent to the Golgin‐positive Golgi apparatus under isotype control conditions. The yellow arrow highlights a representative accumulation of OMP‐positive puncta in the perinuclear region. In contrast, TLR2 neutralization markedly reduced OMV‐associated fluorescence and largely abolished the perinuclear OMP signal.

Ponceau S staining of the membranes, together with subsequent immunoblot analysis, confirmed that OMVs derived from wild‐type and Δ*napA* strains contained comparable levels of a 17‐kDa outer membrane protein (OMP) and the urease subunit UreB (Figure 7C), indicating comparable protein loading between samples. These results suggest that deletion of *napA* does not substantially alter the abundance of these vesicular components, although changes in other OMV‐associated factors cannot be ruled out. Together, these controls indicate that the reduced cellular association observed for Δ*napA* OMVs is unlikely to reflect differences in protein loading or the abundance of these major OMV‐associated proteins

We next examined whether NapA contributes to this TLR2‐dependent association by comparing OMVs derived from the wild‐type strain with those from its isogenic Δ*napA* mutant. In line with the reduced signalling capacity observed for Δ*napA* OMVs in previous experiments, high‐magnification fluorescence microscopy revealed a distinct punctate pattern of DiR‐labeled OMVs (red) decorating the plasma membrane of TLR2‐expressing cells. This physical docking was markedly reduced in Δ*napA* OMVs, supporting the conclusion that OMV‐associated NapA contributes to efficient OMV association with the host cell surface, where NapA‐deficient OMVs associated less efficiently with both TLR2/1‐ and TLR2/6‐expressing AGS cells than wild‐type OMVs (Figure 7D). This difference was further supported by quantitative fluorescence analysis, which revealed a significant decrease in the OMV‐associated signal in cells exposed to Δ*napA* OMVs (Figure 7E), suggesting that NapA promotes efficient OMV association with TLR2‐expressing cells.

To corroborate these fluorescence‐based observations using an independent biochemical approach, we next assessed the presence of OMV‐derived bacterial proteins within host cells following OMV exposure. Consistent with the microscopy data, immunoblot analysis of protein extracts from TLR2/6‐expressing AGS cells stimulated for 5 h with increasing OMV concentrations revealed higher levels of OMV‐derived proteins, including OMP and UreB, in cells exposed to wild‐type OMVs compared with Δ*napA* OMVs, while cellular β‐actin levels remained unchanged (Figure 7F). We next examined the cellular distribution of OMVs by immunofluorescence microscopy following TLR2 neutralization. Under isotype control conditions (control IgA), OMV‐derived OMP immunofluorescence was readily detected at the cell surface and as discrete puncta within the perinuclear region adjacent to the Golgin‐positive Golgi apparatus. In contrast, TLR2 neutralization markedly reduced OMV‐associated fluorescence and substantially diminished the perinuclear OMP signal (Figure 7G). These findings indicate that functional TLR2 is required for efficient cellular association of *H. pylori* OMVs. Moreover, the concomitant reduction in the perinuclear OMV signal following TLR2 neutralization suggests that TLR2‐dependent OMV association represents an early event, thereby facilitating the subsequent intracellular localization of a subset of OMVs (Triantafilou et al. 2006).

Collectively, these findings support a model in which NapA enhances the physical association of *H. pylori* OMVs with TLR2‐expressing epithelial cells, thereby promoting subsequent TLR2‐dependent signalling rather than acting solely as a soluble pro‐inflammatory ligand.

### 3.8 NapA‐Driven OMV‐Induced NF‐κB Activation Is Attenuated by the Natural Compound Sulforaphane

Given the robust activation of the TLR2/NF‐κB axis elicited by *H. pylori* OMVs, we next evaluated whether this inflammatory signalling could be pharmacologically modulated using a natural compound. To this end, AGS cells expressing TLR2/6 were pre‐treated with sulforaphane prior to stimulation with wild‐type *H. pylori* 26695 OMVs (10 µg/mL) or TNF (20 ng/mL) as a control. Pre‐treatment with sulforaphane (10 µM) markedly attenuated OMV‐ and TNF‐induced NF‐κB reporter activity, reducing it to levels comparable to unstimulated controls, indicating effective inhibition of NF‐κB signalling (Figure 8A). Sulforaphane alone did not induce NF‐κB activation, excluding nonspecific proinflammatory effects under the conditions tested.

**FIGURE 8:**
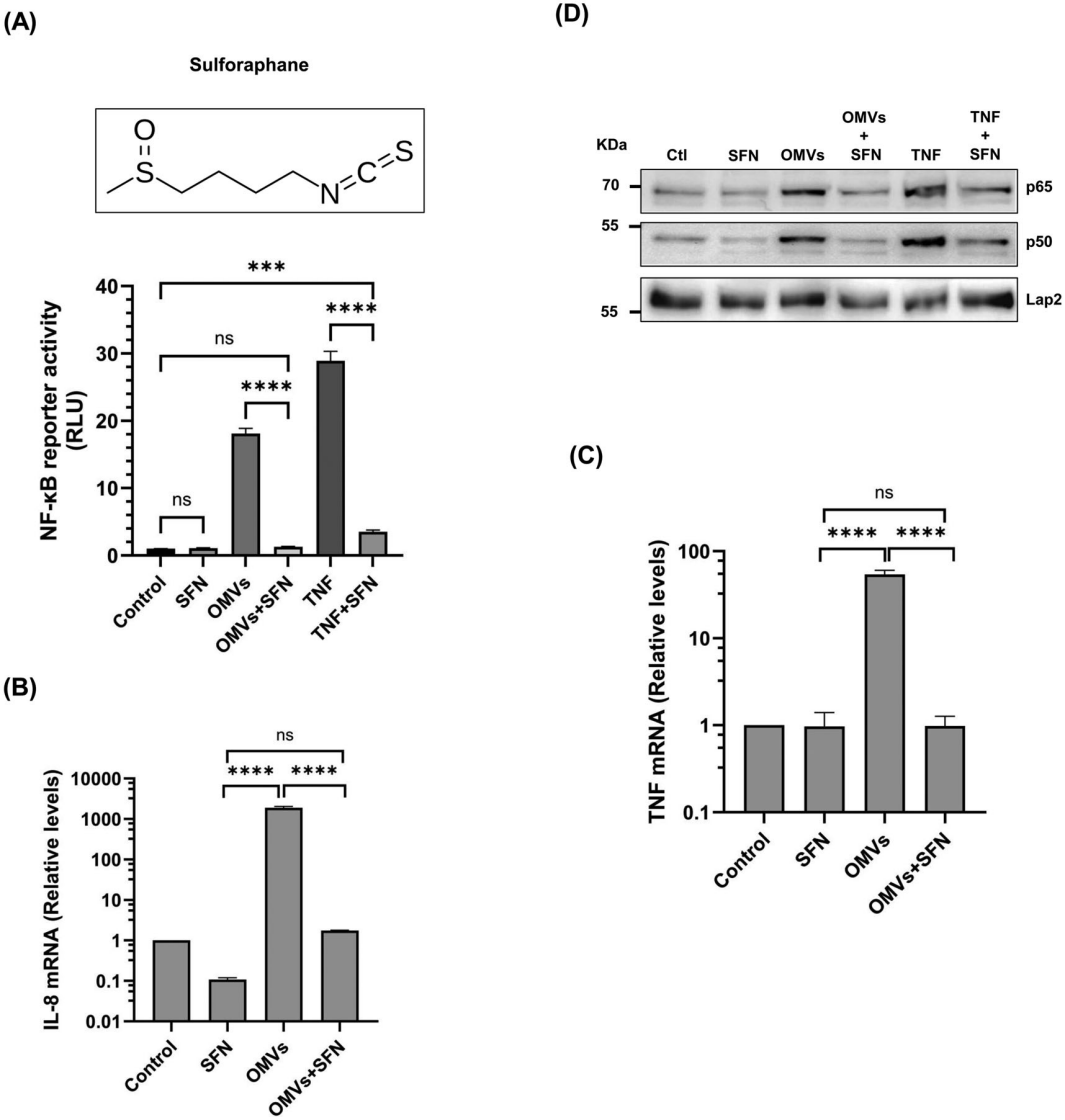
Sulforaphane attenuates OMV‐induced NF‐κB activation and proinflammatory gene expression in gastric epithelial cells. (A) NF‐κB reporter activity in AGS cells stably expressing TLR2/6 pre‐treated with sulforaphane (SFN, 10 µM) prior to stimulation with OMVs derived from *H. pylori* 26695 (WT; 10 µg/mL) for 5 h. NF‐κB–dependent luminescent reporter activity was measured after stimulation and expressed relative to untreated controls as relative light units (RLU). Bars represent the mean ± SD (*n* = 3). (B, C) Relative mRNA expression levels of IL‐8 (B) and TNF (C) in TLR2/6‐expressing AGS cells pre‐treated with sulforaphane and stimulated with WT OMVs for 5 h. Gene expression levels were determined by RT–qPCR, normalized to housekeeping genes, and expressed relative to untreated controls. Bars represent the mean ± SD (*n* = 3). Statistical significance is indicated as ***p < 0.001 and ****p < 0.0001; ns, not significant. (D) Immunoblot analysis of nuclear fractions from TLR2/6‐expressing AGS cells stimulated for 45 min with WT OMVs (10 µg/mL) or TNF (20 ng/mL), in the presence or absence of sulforaphane (10 µM). LAP2 was used as a marker for the nuclear fraction. Blots are representative of three independent experiments.

Consistent with the reporter assay results, sulforaphane strongly suppressed OMV‐induced IL‐8 and TNF mRNA expression in TLR2/6‐expressing AGS cells (Figure 8B,C), supporting inhibition of downstream proinflammatory gene expression.

To further validate that the effects observed in reporter activity and cytokine expression reflected bona fide inhibition of the NF‐κB pathway, we next examined the nuclear translocation of endogenous NF‐κB subunits. Since nuclear accumulation of p65 and p50 represents a hallmark of canonical NF‐κB activation, cytoplasmic and nuclear fractionation assays were performed in TLR2/6‐expressing AGS cells following OMV stimulation. Stimulation with wild‐type *H. pylori* 26695 OMVs (10 µg/mL) induced robust accumulation of both p65 and p50 in nuclear fractions within 45 min, comparable to that observed upon TNF stimulation (Figure 7D). Importantly, pre‐treatment with sulforaphane markedly reduced OMV‐ and TNF‐induced nuclear accumulation of p65 and p50, indicating inhibition of NF‐κB activation at or upstream of nuclear translocation (Figure 8D). Fraction purity was confirmed by enrichment of LAP2 in nuclear fractions.

Together, these data demonstrate that *H. pylori* OMVs induce functional NF‐κB activation in gastric epithelial cells and that this response can be effectively attenuated by sulforaphane.

## 4 Discussion

OMVs released by *H. pylori* are increasingly recognized as key mediators of host–pathogen communication (Jarzab et al. 2020, Wang et al. 2024). However, the molecular mechanisms underlying their initial interactions with host cells and the subsequent activation of innate immune signalling remain incompletely understood. In the present study, we provide mechanistic evidence that NapA, when associated with OMVs, serves as a major determinant of TLR2‐dependent OMV association with gastric epithelial cells, thereby promoting activation of the NF‐κB pathway under the experimental conditions evaluated. These findings support the concept that *H. pylori* OMVs act as organized signalling platforms rather than passive carriers of bacterial components, whose vesicle‐associated presentation of virulence factors critically shapes receptor engagement and downstream inflammatory signalling.

Our findings demonstrate that *H. pylori* OMVs efficiently interacted with gastric epithelial cells expressing TLR2/1 or TLR2/6, resulting in robust NF‐κB activation. Comparative analysis of multiple isogenic mutant strains identified NapA as the predominant OMV‐associated determinant of this response under the experimental conditions evaluated. Notably, deletion of *napA* markedly impaired OMV association and reduced the induction of the pro‐inflammatory cytokines IL‐8 and TNF, supporting a central role for vesicle‐associated NapA during the earliest stages of host‐cell interaction. By contrast, although urease contributed modestly to inflammatory signalling at higher OMV concentrations, its absence had little effect on OMV association, suggesting that urease modulates downstream signalling rather than the initial interaction between OMVs and epithelial cells.

NapA has long been recognized as an immunomodulatory virulence factor of *H. pylori*, predominantly investigated in its soluble form, and has been shown to stimulate cytokine production, promote chemotaxis and activate NF‐κB‐dependent signalling pathways (D'Elios et al. 2007; Amedei et al. 2006). However, although NapA has been identified as a component of *H. pylori* OMVs, considerably less is known about how its vesicular presentation influences its biological activity during host–pathogen interactions (Palacios et al. 2023; Turner et al. 2018). Our findings help address this gap by showing that OMV‐associated NapA promotes TLR2‐dependent OMV association with gastric epithelial cells and elicits a stronger TLR2‐dependent inflammatory response than recombinant NapA under the experimental conditions evaluated. These observations suggest that the biological activity of NapA is influenced not only by its intrinsic immunostimulatory properties but also by its mode of presentation. Within the structural context of OMVs, NapA appears to facilitate TLR2‐dependent OMV association during the earliest stages of OMV–host cell interaction, supporting a model in which vesicular presentation shapes host innate immune recognition. More broadly, these findings suggest that the biological activity of bacterial virulence factors should be interpreted in the context of their structural presentation to host cells (Jarzab et al. 2020; Wang et al. 2024). While studies using purified bacterial components have been instrumental in defining receptor specificity and signalling mechanisms, our results indicate that their relative biological contribution during OMV‐mediated host interactions may be substantially influenced by their native vesicular organization.

Microscopy analyses provided complementary evidence supporting the role of vesicle‐associated NapA by demonstrating a marked reduction in OMV‐associated fluorescence in cells exposed to Δ*napA* OMVs. Although these experiments were not designed to unequivocally distinguish surface‐bound from internalized vesicles, the predominantly punctate and perinuclear distribution of fluorescence observed following stimulation with wild‐type OMVs, together with its apparent proximity to the Golgin‐positive compartment, is compatible with early events occurring following OMV association with host cells. Importantly, TLR2 neutralization markedly altered this distribution pattern, largely abolishing the characteristic perinuclear accumulation observed with wild‐type OMVs, while also reducing OMV‐associated fluorescence to levels comparable to those observed with Δ*napA* OMVs. These observations complement the functional data and support the conclusion that vesicle‐associated NapA facilitates the earliest stages of TLR2‐dependent OMV–host cell interaction. Together, these findings suggest that NapA contributes to the establishment of early OMV–host cell interactions before the initiation of downstream inflammatory signalling. These observations are consistent with previous evidence showing that TLR2 can contribute to early host–pathogen interactions beyond canonical inflammatory signalling, including bacterial adhesion and invasion into epithelial cells in other bacterial systems (Ding et al. 2024).

The contribution of TLR2 to *H. pylori* pathogenesis remains incompletely understood and appears to be highly context‐dependent (Rad et al. 2009; Gringhuis et al. 2014; Sun et al. 2013; Banerjee et al. 2014; Kumar Pachathundikandi et al. 2015). Although TLR2 signalling is generally associated with bacterial recognition and pro‐inflammatory responses, increasing evidence indicates that it may also contribute to immune regulation and tissue homeostasis depending on the receptor complex engaged, the nature of the bacterial ligand, and the responding cell type. This context dependency likely explains the apparently conflicting roles attributed to TLR2 during *H. pylori* infection. Rather than functioning as a single signalling receptor, TLR2 forms heterodimeric complexes with TLR1, TLR6 and potentially TLR10, each of which has been associated with distinct ligand preferences and downstream biological outcomes, ultimately leading to either pro‐inflammatory or immunoregulatory responses depending on the biological context. Our findings build upon this framework by identifying vesicle‐associated NapA as a major determinant of TLR2‐dependent OMV–host cell interactions under the experimental conditions examined.

The potential contribution of *H. pylori* LPS to TLR2 activation has been the subject of considerable debate (Yokota et al. 2007; Smith et al. 2017). However, two independent lines of evidence obtained in this study do not support LPS as the major determinant of the TLR2‐dependent responses observed under our experimental conditions. First, purified *H. pylori* LPS elicited markedly weaker NF‐κB activation than intact OMVs, despite being tested at elevated Kdo‐normalized concentrations. Second, disruption of LPS glycan biosynthesis through deletion of *waaL* or *lex2B* produced only modest effects on OMV‐induced NF‐κB activation while preserving the strong phenotype associated with *napA* deletion. Collectively, these findings indicate that although LPS may contribute to TLR2 signalling, alterations in LPS structure alone are insufficient to account for the marked reduction in OMV activity caused by *napA* deletion. Instead, these results identify vesicle‐associated NapA as a major determinant of TLR2‐dependent OMV–host cell interactions under the experimental conditions evaluated. Taken together, these findings suggest that the relative contribution of individual bacterial ligands cannot be explained solely by their intrinsic receptor‐binding properties, but must also be interpreted within their native structural context (Triantafilou et al. 2006; Jarzab et al. 2020).

Although our results identify TLR2 as a major mediator of NapA‐dependent OMV recognition, they do not imply that NapA is the only *H. pylori* component capable of engaging this receptor. Several bacterial molecules, including urease, Hsp60 and LPS, have previously been reported to activate TLR2‐dependent signalling (Valenzuela‐Valderrama et al. 2019; Gringhuis et al. 2014; Smith et al. 2017; Smith 2014; Gonzalez‐Lopez et al. 2013; Muotiala et al. 1992; Alvarez‐Arellano et al. 2007). Our findings extend these observations by demonstrating that the relative contribution of individual TLR2 agonists is strongly influenced by the structural context in which they are presented, with vesicle‐associated NapA emerging as the major determinant of TLR2‐dependent OMV–host interactions under the experimental conditions evaluated. At the same time, our results do not exclude contributions from additional pattern recognition receptors. Several receptors have been implicated in host responses to *H. pylori*, including TLR4, TLR5, TLR9 and the cytosolic receptor NOD1 (Kaparakis et al. 2010; Smith 2014). In gastric epithelial cells, NOD1 is a well‐established sensor of peptidoglycan delivered through the type IV secretion system (Viala et al. 2004), whereas the expression, subcellular localization and responsiveness of TLR4, TLR5 and TLR9 appear to be more variable and context‐dependent (Schmausser et al. 2004; Castano‐Rodriguez et al. 2014). The minimal OMV‐induced NF‐κB activation observed in mock‐control AGS cells is therefore unlikely to reflect a complete absence of alternative sensing pathways. Rather, it suggests that, under our experimental conditions, these receptors do not efficiently compensate for the absence of TLR2 heterodimers during the earliest stages of OMV–host cell interaction. Because the present study focused primarily on these early events, the relative contribution of intracellular pattern recognition receptors and their integration with TLR2 signalling remains to be determined. Endosomal receptors such as TLR9 may also participate following OMV uptake and intracellular processing, while the coordinated integration of multiple signalling pathways is likely to influence the magnitude and quality of host inflammatory responses during infection. Accordingly, the mechanism proposed here should be interpreted as one important component of a broader and more complex network of OMV–host interactions.

The identification of vesicle‐associated NapA as a major determinant of early OMV–host cell interactions has broader implications for understanding *H. pylori* pathogenesis. Chronic exposure of the gastric mucosa to OMVs may continuously reinforce low‐grade inflammatory signalling, thereby contributing to persistent epithelial activation and disease progression. By facilitating efficient host‐cell recognition during the earliest stages of OMV interaction, NapA may amplify persistent inflammatory responses over prolonged infection. Such a mechanism may contribute to the persistent inflammatory microenvironment that favours gastric carcinogenesis. The ability of sulforaphane to suppress NapA‐dependent NF‐κB activation further supports the functional relevance of this signalling pathway. Sulforaphane has previously been reported to exert anti‐inflammatory effects through multiple mechanisms, including inhibition of IKK activation, suppression of IκBα phosphorylation and degradation, reduced NF‐κB nuclear translocation, and activation of the Nrf2 antioxidant pathway, all of which converge to attenuate pro‐inflammatory gene expression (Habtemariam 2024; Xu et al. 2005). Although the precise mechanism underlying the inhibitory effect observed in the present study was not directly investigated, the reported ability of sulforaphane to inhibit NF‐κB signalling downstream of receptor activation is consistent with our observations. Our findings therefore suggest that sulforaphane primarily attenuates host inflammatory signalling elicited by vesicle‐associated NapA rather than interfering with the earliest stages of OMV association.

Several limitations should be acknowledged. Although our findings consistently identify vesicle‐associated NapA as a major determinant of OMV‐induced TLR2 activation under the experimental conditions evaluated, the precise molecular interface between NapA and TLR2 remains to be defined. In addition, the experiments were performed primarily in engineered gastric epithelial cell models designed to isolate TLR2‐dependent signalling and therefore may not fully capture the complexity of host responses observed during natural infection. Future studies using physiologically relevant infection models will help determine how NapA cooperates with additional bacterial and host factors to regulate OMV‐mediated pathogenesis in vivo.

Based on these findings, we propose a working model in which vesicle‐associated NapA promotes the earliest stages of TLR2‐dependent OMV association with gastric epithelial cells, thereby enhancing NF‐κB activation and local inflammatory responses. As illustrated in Figure 9, this mechanism may also provide a conceptual framework for understanding how *H. pylori* OMVs could contribute to systemic host responses following dissemination beyond the gastric mucosa.

**FIGURE 9:**
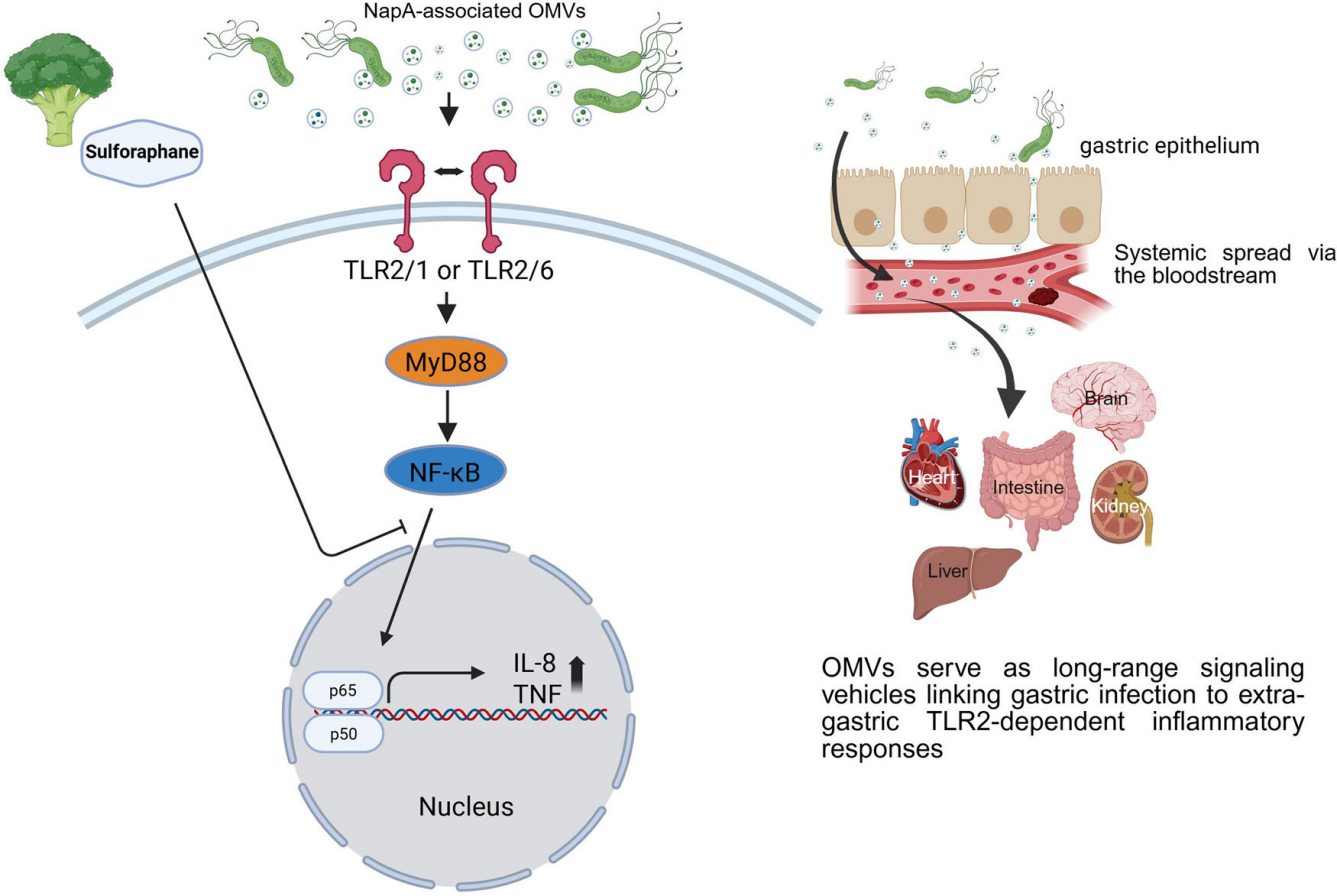
Proposed model of TLR2–NF‐κB activation mediated by NapA‐associated *Helicobacter pylori* OMVs and their potential systemic impact. OMVs released by *Helicobacter pylori* act as organized signalling platforms that modulate both local and potentially systemic inflammatory responses. In this model, Neutrophil‐Activating Protein A (NapA), associated with the OMV surface, promotes the interaction and clustering of TLR2 heterodimers (TLR2/1 or TLR2/6) on gastric epithelial cells. This spatial organization enhances signalling efficiency compared with soluble NapA, leading to activation of the canonical MyD88‐dependent pathway and nuclear translocation of NF‐κB (p50/p65 heterodimer). Locally, NF‐κB activation induces the transcription of pro‐inflammatory mediators, including IL‐8 and TNF. The natural compound sulforaphane negatively modulates this pathway by inhibiting NF‐κB activation, as indicated by the flat‐ended inhibitory arrow. In addition to local epithelial signalling, NapA‐enriched OMVs may traverse the gastric epithelial barrier and enter the systemic circulation, thereby facilitating the dissemination of bacterial components to distant organs, including the liver, heart, brain, intestine, and kidney. This integrative model provides a mechanistic framework to explain how *H. pylori* OMVs may contribute to extra‐gastric inflammatory responses associated with chronic infection. IL‐8 indicates interleukin‐8; MyD88 myeloid differentiation primary response 88; NF‐κB, nuclear factor kappa‐light‐chain‐enhancer of activated B cells; OMV outer membrane vesicle; TLR2, Toll‐like receptor 2; TNF, tumour necrosis factor (Figure created in https://BioRender.com).

In conclusion, our study identifies vesicle‐associated NapA as a major determinant of the earliest stages of OMV–host cell interactions. By promoting efficient TLR2‐dependent OMV association with gastric epithelial cells, vesicle‐associated NapA facilitates downstream NF‐κB activation and inflammatory signalling. These findings identify vesicle‐associated NapA as a previously unrecognized major determinant of TLR2‐dependent innate immune recognition of *H. pylori* OMVs, while supporting the contribution of additional OMV‐associated components to the overall host response.

## Author Contributions

**Mariaignacia Rubilar**: investigation, methodology, validation, formal analysis, software, data curation. **Rosy Brito**: investigation, methodology, supervision. **Annegrett Palavecino**: methodology, data curation. **Joan Villena**: investigation, data curation. **Marco Mellado**: investigation, methodology, formal analysis. **María I. Silva**: investigation, data curation. **Manuel Valenzuela‐valderrama**: conceptualization, investigation, funding acquisition, writing – original draft, methodology, validation, visualization, writing – review and editing, software, formal analysis, project administration, data curation, supervision, resources.

## Funding

This work was funded by the grant ANID Fondecyt Regular #1230590 (M.V.‐V.) and Fondo Concursable de Fomento de la Productividad Científica UCEN 2026, Grant #CIFP202607 (M.V.‐V.).

## Conflicts of Interest

The authors declare no conflicts of interest.

## Data Availability

Any additional information required to reanalyze the data reported in this paper is available from the authors upon request.
